# Translational PK/PD and the first-in-human dose selection of a PD1/IL15: an engineered recombinant targeted cytokine for cancer immunotherapy

**DOI:** 10.3389/fphar.2024.1380000

**Published:** 2024-06-03

**Authors:** Rajbharan Yadav, Suzanne Schubbert, Patrick G. Holder, Eugene Y. Chiang, Nargess Kiabi, Liz Bogaert, Irene Leung, Rumana Rashid, Kendra N. Avery, Christine Bonzon, John R. Desjarlais, Shomyseh Sanjabi, Amy Sharma, Michelle Lepherd, Amy Shelton, Pam Chan, Yanqiu Liu, Louis Joslyn, Iraj Hosseini, Eric G. Stefanich, Amrita V. Kamath, Matthew J. Bernett, Vittal Shivva

**Affiliations:** ^1^ Genentech, Inc., South SanFrancisco, CA, United States; ^2^ Xencor, Inc., Pasadena, CA, United States

**Keywords:** targeted cytokine, PD1/IL15 TaCK, minimum anticipated biological effect level, first-in-human dose, target mediated drug disposition

## Abstract

**Introduction:**

Interleukin 15 (IL-15) is a potential anticancer agent and numerous engineered IL-15 agonists are currently under clinical investigation. Selective targeting of IL-15 to specific lymphocytes may enhance therapeutic effects while helping to minimize toxicities.

**Methods:**

We designed and built a heterodimeric targeted cytokine (TaCk) that consists of an anti-programmed cell death 1 receptor antibody (anti-PD-1) and an engineered IL-15. This “PD1/IL15” selectively delivers IL-15 signaling to lymphocytes expressing PD-1. We then investigated the pharmacokinetic (PK) and pharmacodynamic (PD) effects of PD1/IL15 TaCk on immune cell subsets in cynomolgus monkeys after single and repeat intravenous dose administrations. We used these results to determine the first-in-human (FIH) dose and dosing frequency for early clinical trials.

**Results:**

The PD1/IL15 TaCk exhibited a nonlinear multiphasic PK profile, while the untargeted isotype control TaCk, containing an anti-respiratory syncytial virus antibody (RSV/IL15), showed linear and dose proportional PK. The PD1/IL15 TaCk also displayed a considerably prolonged PK (half-life range ∼1.0–4.1 days) compared to wild-type IL-15 (half-life ∼1.1 h), which led to an enhanced cell expansion PD response. The PD was dose-dependent, durable, and selective for PD-1^+^ lymphocytes. Notably, the dose- and time-dependent PK was attributed to dynamic TMDD resulting from test article-induced lymphocyte expansion upon repeat administration. The recommended first-in-human (FIH) dose of PD1/IL15 TaCk is 0.003 mg/kg, determined based on a minimum anticipated biological effect level (MABEL) approach utilizing a combination of *in vitro* and preclinical *in vivo* data.

**Conclusion:**

This work provides insight into the complex PK/PD relationship of PD1/IL15 TaCk in monkeys and informs the recommended starting dose and dosing frequency selection to support clinical evaluation of this novel targeted cytokine.

## Introduction

Cytokines play an important role in the immune response to cancer and are being tested as potential therapeutics either alone or in combination with other agents (Atallah-Yunes and Robertson, 2022). A particular focus has been on cytokines that can augment T cell- and NK cell-mediated cancer immunity. Recombinant interleukin 2 (IL-2, aldesleukin) was approved for the treatment of metastatic melanoma and renal cell carcinoma over two decades ago (Noble and Goa, 1997; Wrangle et al., 2018). IL-2 is thought to drive anti-tumor immunity by enhancing the proliferation, survival, and effector function of lymphocytes that express the heterodimeric IL-2/IL-15 receptor (IL-2/15Rβ/common γ, CD122/CD132, Rβγ) (Waldmann et al., 1998; Waldmann, 2006), such as NK cells, activated T cells, and resting memory CD8^+^ T cells (Dubois et al., 2002). Despite its benefit to a subset of patients, the use of IL-2 is limited by its toxicity profile and short half-life, requiring intensive inpatient monitoring during and after administration. IL-2 signaling through Rβγ is enhanced as a result of capture of IL-2 by the private IL-2 receptor α (CD25; IL-2Rα) subunit. This capture of IL-2 by CD25 is vital for the endogenous anti-tumor and anti-viral activity of activated CD8^+^CD25^+^ cells (Hashimoto et al., 2022). However, when IL-2 is administered recombinantly in high dose, it can also lead to undesirable downstream effects on CD25^hi^ cells, including amplification of CD4^+^ regulatory T cell (T_reg_) populations, activation of endothelial cells leading to vascular leak, and an autocrine feedback loop on activated T cells leading to activation-induced cell death (AICD) (Schwartz et al., 2002; Waldmann, 2015).

Similar to IL-2, IL-15 is a member of the common γ family and drives proliferation and increased function of effector lymphocytes; in contrast, IL-15 interacts via its distinct IL-15 receptor α (CD215; IL-15Rα), resulting in a superior safety profile over IL-2. This includes a lower incidence of vascular leak, reduced T_reg_ expansion, and lack of AICD. Recombinant human IL-15 (rhIL-15) has been tested in Phase I clinical trials (Conlon et al., 2015; Miller et al., 2018; Conlon et al., 2019a; Conlon et al., 2019b). When administered as an intravenous (IV) bolus, rhIL-15 was cleared rapidly *in vivo*, with a short half-life in humans (∼1–3 h) and transient PD effects (Waldmann et al., 2011; Conlon et al., 2015). To enable sustained pharmacological effects, rhIL-15 required high and frequent doses which resulted in substantial PK fluctuations and elevated peak concentrations, leading to dose-limiting toxicities and other adverse events (Conlon et al., 2015; Margolin et al., 2018; Romee et al., 2018; Conlon et al., 2019a; Conlon et al., 2019b). Alternative dosing approaches such as subcutaneous (SC) dosing and continuous IV infusion were investigated, but also presented unique challenges including injection site reactions and logistical complexity (Conlon et al., 2015; Margolin et al., 2018; Romee et al., 2018; Conlon et al., 2019a; Conlon et al., 2019b). Engineered and half-life extended cytokines (Margolin et al., 2018; Bernstein and Spangler, 2021; Alonso Fernández et al., 2022) may enable the development of IL-15 therapeutics with improved PK profiles and therapeutic indices. Recent approaches involve targeted delivery of IL-15 to T cells that may be enriched for antigen specificity capable of mediating anti-tumor immunity, thereby enhancing the therapeutic benefit of IL-15 based therapies (Martomo et al., 2021; Xu et al., 2021; Shen et al., 2022).

**FIGURE 1:**
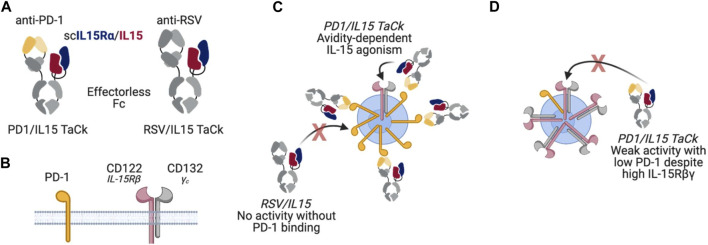
**(A)** PD1/IL15 and untargeted control RSV/IL15 molecule design **(B)** PD1 and IL-15βγ receptor subunits **(C)** PD1/IL15 TaCk avidity dependent IL-15 agonism; For RSV/IL15: No activity without PD-1 binding **(D)** PD1/IL15 TaCk demonstrates weak activity with low PD-1 despite the high IL-15βγ receptors.

In this study, we combined an engineered variant of IL-15 and an anti-programmed cell death protein 1 antibody (anti-PD-1) into PD1/IL15, a recombinant targeted cytokine (TaCk). The PD1/IL15 TaCk is a single heterodimeric, effectorless antibody fragment crystallizable (Fc) fusion (Figure 1). The first component is a humanized IgG1 half-antibody (Ab) that binds to PD-1, but does not block the PD-1/PD-L1 interaction. The second component is the extracellular sushi domain of IL-15Rα fused to an engineered variant of human IL-15 to generate a single chain (scIL-15Rα/IL-15). This domain of PD1/IL15 selectively engages the Rβγ complex. The scIL-15Rα/IL-15 is engineered to have extremely weak affinity toward Rβγ, such that it requires PD-1 binding through the anti-PD-1 to elicit Rβγ signaling by avidity. This is expected to result in targeted agonism of Rβγ only on PD-1^+^ lymphocytes, leading to selective expansion and enhanced effector function of these lymphocytes including activated CD8^+^ and CD4^+^ T cells. Additional engineering of the Fc domain of the TaCk include mutations to enhance neonatal FcR (FcRn) binding at lower pH (6.0) to increase half-life, and removal of effector function by mutations that prevent binding to Fcγ and complement receptors. PD1/IL15 does not cross react with murine PD-1 and has extremely weak potency towards mouse IL-2/15Rβγ. Thus, non-clinical PK/PD studies with PD1/IL15 TaCk were limited to the cynomolgus monkeys (monkeys).

Here, we characterized the PK/PD behavior of PD1/IL15 TaCk in monkeys following single- and repeat dose IV administration. We also determined the effect of PD1/IL15 TaCk on multiple PD-1-expressing immune cell subtypes, in particular circulating CD8^+^, CD4^+^, CD8–CD4– (double negative, DN), and regulatory T cells (CD4^+^FoxP3^+^CD25^+^, T_regs_). An isotype control TaCk containing an anti-respiratory syncytial virus (RSV) antibody (RSV/IL15) in place of anti-PD-1 was used to confirm the selective and avidity-driven targeting of PD1/IL15 on PD-1^+^ lymphocytes. Finally, we leveraged *in vitro* pharmacology assays and PK/PD in cynomolgus monkeys to recommend starting dose and dosing frequency for the first-in-human (FIH) clinical trial.

## Materials and methods

### Biacore binding affinity

Binding of PD1/IL15 and RSV/IL15 to human PD-1 antigen were determined by surface plasmon resonance (SPR) using a Biacore T200 (GE Healthcare, Chicago, IL, UnitedSA). Each molecule was diluted to 1 μg/mL in HBS-P running buffer (Cytiva) and captured for 1 min on a Series S Protein A chip (Cytiva). A 100, 20, 4, and 0.8 nM dilution series of either human PD-1 was produced in HBS-P and subsequently flowed across the surface for 5 min followed by 5 min of buffer to quantify the rate of dissociation. All steps were run at 25 °C with a flow rate of 30 μL/min. The Biacore Evaluation Software was used to fit each sensorgram to the nearest kinetic model and obtain affinity measurements.

### *In vitro* potency of PD1/IL15 TaCk on stimulated human PBMCs

The *in vitro* potency of PD1/IL15 TaCk was determined using a proliferation assay on activated PBMC from eight human donors. PBMC were activated with 25 ng/mL plate-bound anti-CD3 for 24 h before the addition of PD1/IL15 TaCk in half-log dilutions from 1 ng/mL to 100 μg/mL. Carboxyfluorescein diacetate succinimidyl ester (CFSE) dilution was used to measure proliferation in T cell subsets after a 4-day treatment with PD1/IL15 TaCk. The potency of PD1/IL15 TaCk to induce proliferation was evaluated in CD4 T cells, CD4 effector memory T cells (CD4^+^T_EM_), CD8 T cells, and CD8 effector memory T cells (CD8^+^T_EM_). Potency was defined in terms of EC_50_, calculated using 4-parameter sigmoidal dose-response curve fits and the least squares method using GraphPad Prism software (La Jolla, California, United States).

$$Y=Bottom+\left(\frac{Top-Bottom}{1+10^{\left(\mathrm{Log}{\text{EC}}_{50}-X\right)-HillSlope}}\right)$$

• Bottom is the Y value at the bottom plateau.
• Top is the Y value at the top plateau.
• LogEC50 is the X value when the response is halfway between Bottom and Top.

Hillslope describes the steepness of the curve. To evaluate the variability in the parameters among the 8 donors and to assess the goodness of fit of the curve, a R squared values for each donor were calculated. In addition, a 95% confidence interval (CI) for Bottom, Top, and EC50 parameters for all 8 donors were calculated to support goodness of fit of curve fitting among all 8 donors. Finally, EC(F) values were determined using following equation, in which H is the HillSlope.

$${EC}_{F}={\left(\frac{F}{100-F}\right)}^{1/H}∙{EC}_{50}$$

### Single and repeat dose cynomolgus monkey studies

PK and PD (absolute counts of lymphocytes) of PD1/IL15 TaCk after single- and repeat-dose administrations were evaluated in two non-[good laboratory practice (GLP)] studies (conducted at Altasciences), and a GLP study (conducted at CRL) in monkeys. In the non-GLP single dose study, eighteen male monkeys were randomly assigned to six groups (n = 3 animals/group) and were administered a single IV dose (0.1, 0.3, or 1 mg/kg) of either PD1/IL15 TaCk or RSV/IL15 (isotype control). Blood samples (0.5 mL) for PK, PD (peripheral blood immunophenotyping) and anti-drug antibodies (ADA) detection were collected from each animal via the femoral vein at designated time points. ADA samples were collected and however, not analyzed for the RSV/IL15. The serum samples from single dose monkey study were analyzed for PD1/IL15 TaCk and RSV/IL15 at Xencor (Monrovia, CA, United States) using a Sandwich DELFIA^®^ TRF method (Peruski et al., 2002) and ADA were characterized using a direct coat ELISA assay. The lower limit of quantification (LLOQ) was 32.8 ng/mL and 97.7 ng/mL for PD1/IL15 TaCk and RSV/IL15 TaCk, respectively.

In the non-GLP repeat dose study, naïve male monkeys (n = 4/dose group) were given slow IV bolus administration of 0.05 mg/kg or 0.6 mg/kg of PD1/IL15 TaCk, once every 2 weeks (Q2W) for a total of three dose cycles (on days 0, 14, and 28). Blood was collected pre-study and at selected time points throughout the study for analyses of toxicokinetics (TK), ADA, and measurement of circulating lymphocyte populations by flow cytometry. An ELISA method was developed and validated to quantify PD1/IL15 in monkey serum samples and a direct coat ELISA method was developed and validated to detect ADA against PD1/IL15 TaCk in monkey serum samples. The LLOQ was 62.5 ng/mL for the non-GLP repeat dose monkey study.

In the GLP study, naive male and female monkeys (total 26 animals) were given slow IV bolus administration of 0.03 mg/kg (n = 6; 3F and 3M), 0.1 mg/kg (n = 10; 5F and 5M) and 0.3 mg/kg (n = 10; 5F and 5M) of PD1/IL15 TaCk, once every 3 week (Q3W) for a total of three dose cycles (on days 0, 21, and 42) followed by an eight-week recovery period. Blood was collected pre-study and at selected time points throughout the study for analyses of TK, ADA, and measurement of circulating lymphocyte populations by flow cytometry. Additional PK, PD and ADA samples from recovery animals (n = 4/group; 2F/2M, out of a total of 10 animals each in 0.1 and 0.3 mg/kg dose groups) were collected for 15 weeks on days 70 and 105 after the third dose of PD1/IL15 TaCk. An ELISA method was developed and validated to quantify PD1/IL15 in monkey serum samples and a bridging ELISA method was developed and validated to detect ADA against PD1/IL15 TaCk in monkey serum samples. The LLOQ in PK assay was 5 ng/mL.

Non-compartmental analysis (NCA) was used to estimate the PK parameters from serum concentration-time profiles obtained in non-GLP and GLP studies, using Phoenix™ WinNonlin^®^, Version 6.4 software (WinNonlin; Certara, Inc., United States, NJ). The complete study details including the design, PK/TK assays/data analyses, ADA assays and immunophenotyping are described in the Supplementary Material. Flow cytometry was used to measure lymphocytes and their subtypes. The cellular antigens and cell populations identified were quantified using the antibody panels and gating strategies shown in Supplementary Table S4 and Supplementary Figure S1.

### First-in-human dose selection of PD1/IL15 TaCk

We considered several approaches and evaluated the totality of data including the non-clinical toxicology, *in vitro* and *in vivo* pharmacology and pharmacokinetics of PD1/IL15 TaCk and compared with the minimal pharmacology active dose (mPAD) identified in humanized murine studies (data not shown). FIH clinical trials require careful selection of a safe yet biologically relevant starting dose. Typically, such starting doses are selected based on toxicity studies in a pharmacologically relevant animal model. However, with the advent of target-specific and highly active immunotherapies, both the Food and Drug Administration (FDA) and the European Medicines Agency (EMA) have provided guidance that recommend determining a safe starting dose based on a minimum anticipated biological effect level (MABEL) approach (Saber et al., 2016; Saber et al., 2017). To recommend a FIH dose for PD1/IL15 TaCk, we ascertained the MABEL based on the most sensitive *in vitro* assays on stimulated hPBMCs and identified the most sensitive and pharmacologically relevant cell subtype that responds to the PD1/IL15 TaCk. To do so, we calculated the concentration of TaCk that resulted in 10, 20, 30, or 50% of the maximal effect (EC_10,_ EC_20,_ EC_30_, or EC_50_) for inducing cell proliferation, measured using CFSE. The EC_50_ value is a conservative measurement of biological activity and can be converted into a FIH dose by equating EC_50_ to the maximum plasma concentration (C_max_) in humans. Thus, the human starting dose was calculated as follows, assuming IV administration to a 70 kg human with physiological volume of distribution of 42.9 mL/kg (Davies and Morris, 1993).

$$FIH\;dose={EC}_{50}∙Human\;Volume\;of\;distribution$$

## Results

### Binding affinity and cross-reactivity

The binding affinity and cross reactivity of PD1/IL15 TaCk and RSV/IL15 towards human PD-1 and mouse PD-1 was assessed by SPR. The results showed robust binding of the anti-human PD-1 arm to human PD-1 with a K_D_ of 3.19 nM, but no cross-reactivity to mouse PD-1 was detected. The RSV control arm did not exhibit any binding to either human or mouse PD-1 (Table 1).

**TABLE 1:** Binding affinities of PD1/IL15 TaCk to human PD-1. PD1/IL15 TaCk EC_50_ (ng/mL; geometric Mean) values for proliferation of activated human PBMC donors (8 donors).

Molecule	Binding affinity (Kd; nM) against human PD-1	*In vitro* EC_50_ (ng/mL) for CD4^+^ and CD8^+^ T cell and their effector memory T cell subsets on stimulated human PBMC			
		CD4 T cells	CD4 T_EM_	CD8 T cells	CD8 T_EM_
PD1/IL15 TaCk^a^	3.19	113.8	69.8	203.1	131.8

^a^ PD1/IL15 TaCk showed no binding to mouse PD-1. NA: IL-15: interleukin 15; TaCk: targeted cytokine; PD-1: programmed cell death 1 receptor; EC_50_: 50% of maximum activity; PBMC: peripheral blood mononuclear cells.

### PD1/IL15 TaCk exhibited greater *in vitro* activity vs. RSV/IL15 on stimulated human PBMC

**FIGURE 2:**
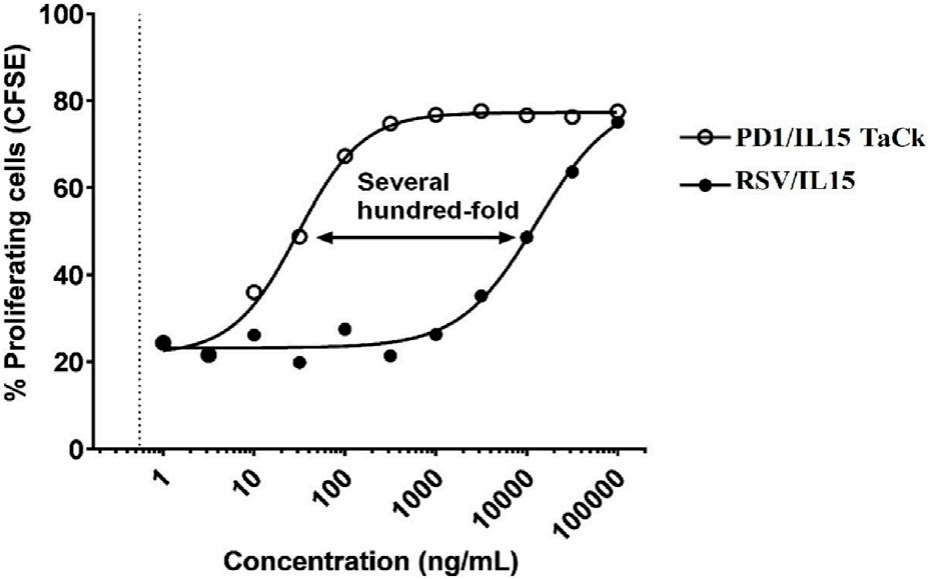
*In vitro* activity of PD1/IL15 TaCk, and untargeted control molecule RSV/IL15 on activated CD8^+^ Effector Memory T cells. CFSE = carboxyfluorescein succinimidyl ester. Human peripheral blood mononuclear cells (PBMC) were stimulated with 500 ng/mL plate-bound anti-CD3 (OKT3) for 48 h then labeled with CFSE, treated with increasing concentrations of PD1/IL15 TaCk or RSV/IL15 control for 4 days at 37 °C, and then analyzed by flow cytometry using CFSE to measure proliferation.

PD1/IL15 TaCk was evaluated in an *in vitro* proliferation assay using human PBMCs stimulated with anti-CD3. Stimulation of the human PBMCs from healthy donors with anti-CD3 is necessary to induce expression of PD-1. PD1/IL15 TaCk has no activity on unstimulated PBMCs whose lymphocytes express much lower levels of PD-1 and CD122 relative to cells after anti-CD3 stimulation. RSV/IL15 contains the same scIL-15Rα/IL-15 arm as PD1/IL15, but substitutes a non-binding anti-RSV arm for the anti-PD-1, and was used as a control to evaluate the activity of the scIL-15Rα/IL-15 independent of the avidity gained through PD-1 binding. The results revealed that the PD1/IL15 TaCk has several hundred-fold higher activity (Figure 2) on activated CD8^+^ T_EM_ cells, which express a high level of PD-1, and CD122 compared to the RSV/IL15. Thus, the selectivity of PD1/IL15 TaCk activity is the result of avidity gained by binding cells expressing both PD-1 and IL-15 Rβγ.

### CD4^+^ effector memory T cells are the most sensitive cell type to PD1/IL15 TaCk treatment in *in vitro* assays

The *in vitro* potency of PD1/IL15 TaCk to induce proliferation was assessed on activated PBMCs from 8 human donors. Various cell subsets such as CD4^+^ T cells, CD4^+^ effector memory T cells (CD4^+^ T_EM_), CD8^+^ T cells, and CD8^+^ effector memory T cells (CD8^+^ T_EM_) cells were evaluated to assess PD1/IL15 TaCk potency. The EC_50_ analysis with 4-parameter sigmoidal dose-response curve fits and the least squares method was used to determine potency, and the results are shown in Table 1. CD4^+^ T_EM_ cells were the most sensitive subset in activated human PBMCs to PD1/IL15 TaCk-induced proliferation. The mean (geometric) potency of PD1/IL15 TaCk to induce proliferation of CD4 ^+^T_EM_ was calculated from individual EC_50_ values from all individual donors and mean EC_50_ was 69.8 ng/mL. Similarly, EC_50_ of CD4^+^ T cells, CD8^+^ T cells and CD8^+^T_EM_ cells were 113.8 ng/mL, 203.1 ng/mL, and 131.8 ng/mL, respectively (Table 1). Other EC values across cell subsets are presented in Supplementary Table S1. While there is some variability in the parameters across the 8 donors, the R-squared values for each donor are close to 100%, indicating a high level of precision and excellent goodness of fit for the curves. Comprehensive details, including 95% confidence intervals for the Bottom, Top, and EC50 parameters for all 8 donors, are provided in Supplementary Table S2. These additional analyses further support the goodness of fit of the curve fitting and highlight the precision observed across all 8 donors.

### PD1/IL15 TaCk exhibited multiphasic non-linear PK behavior impacted by target-mediated drug disposition while RSV/IL15 showed biphasic dose proportional PK in cynomolgus monkeys

**FIGURE 3:**
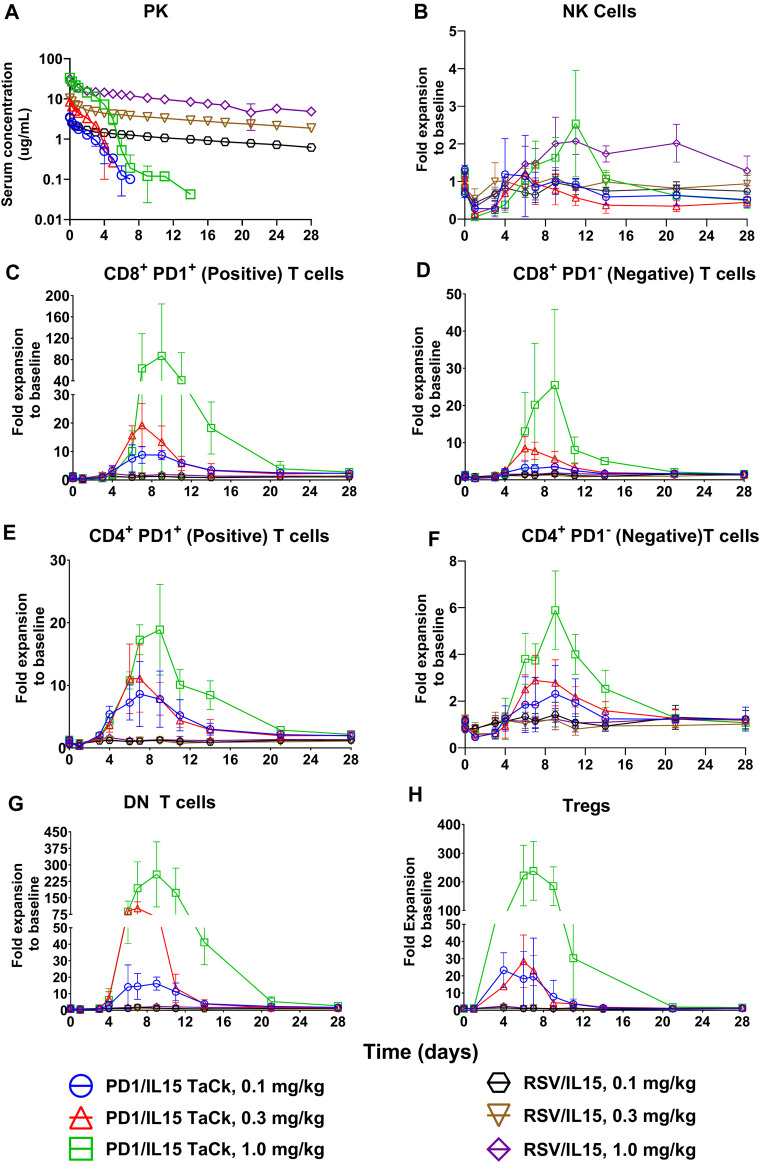
PK profiles **(A)** of PD1/IL15 TaCk, and RSV/IL15 and dose dependent PD-1^+^ lymphocyte expansion **(B–H)** by PD1/IL15 TaCk with selectivity over isotype control RSV/IL15 following a single IV bolus administration in cynomolgus monkeys (n = 3/group); [three dose groups: 0.1 mg/kg, 0.3 mg/kg, and 1 mg/kg]. The data are represented as mean ± SD. Note: a significant number of PK concentration data points were below limit of quantification (<32.8 ng/mL) in all PD1/IL15 TaCk treatment groups (8, 10 and 6 time points for 0.1, 0.3 and 1 mg/kg, respectively) and were neither presented in the figure nor used in NCA PK parameter estimation. Bolds are Doses it is there is left column it is mentioned in first column, thus no change needed.

PK and PD of the PD1/IL15 TaCk and RSV/IL15 were characterized in monkeys following a single IV bolus of 0.1, 0.3, or 1 mg/kg (Figure 3). PK parameters estimated using Non-compartmental analysis (NCA) are summarized in Table 2. PD1/IL15 TaCk exhibited a multiphasic PK profile with the mean CL ranging from 14.0 to 20.6 mL/day/kg, and mean V_ss_ in the range of 26.9–32.7 mL/kg following single-dose IV administration. The PK profiles showed an increase in systemic exposure (C_max_ and AUC_0-∞_) that was slightly less than dose proportional between 0.1 and 0.3 mg/kg, and greater than dose proportional between 0.3 and 1 mg/kg. In general, the observed C_max_ was slightly higher at all doses of PD1/IL15 TaCk than expected based on blood volume of 40–50 mL/kg in cynomolgus monkeys (Davies and Morris, 1993). The CL of PD1/IL15 TaCk was higher than normal IgG CL (<8 mL/d/kg) (Hötzel et al., 2012) and was dose-dependent, suggesting nonlinear PK. On the other hand, RSV/IL15 demonstrated biphasic dose proportional PK characterized by a rapid initial distribution phase followed by a slower elimination phase. The mean CL ranged from 2.05 to 3.07 mL/day/kg and V_ss_ was in the range of 53.5–59.3 mL/kg following single-dose IV administration of RSV/IL15. All animals (100%) dosed with PD1/IL15 TaCk were ADA positive. Specifically, animals in mid and high dose groups (0.3 mg/kg and 1 mg/kg) had high ADA titers (ranging from 3.5 to 4.3) starting from day 14, while animals in the low dose group (0.1 mg/kg) had relatively low ADA titers ranging from 2.0 to 2.5. Additionally, all pre-dose samples were ADA negative with a titer of <1.70 (Supplementary Table S3).

**TABLE 2:** Summary of Mean (±SD) PK parameters following a single IV administration of PD1/IL15 TaCk and RSV/IL15 (isotype control) in cynomolgus monkeys.

PK parameters	PD1/IL15 TaCk			RSV/IL15		
Dose (mg/kg)	**0.1**	**0.3**	**1**	**0.1**	**0.3**	**1**
C_max_ (µg/mL)	3.49 ± 0.62	8.5 ± 0.7	33.5 ± 1.01	3.44 ± 0.31	10.3 ± 1.13	31.5 ± 7.4
Dose normalized C_max_ (C_max_/Dose)	34.9 ± 6.2	28.3 ± 2.3	33.5 ± 1.01	34.4 ± 3.1	34.3 ± 3.8	31.5 ± 7.4
AUC_0-∞,_ (day•µg/mL)	6.44 ± 1.53	14.8 ± 2.2	71.3 ± 2.26	48.8 ± 1.9	145 ± 9.96	339 ± 75.9
Dose normalized AUC (AUC_0-∞_/Dose)	64.4 ± 15.3	49.3 ± 7.3	71.3 ± 2.26	488 ± 19	483 ± 33.2	339 ± 75.9
CL (mL/day/kg)	16.2 ± 1.02	20.6 ± 2.93	14 ± 0.43	2.05 ± 0.08	2.08 ± 0.14	3.07 ± 0.79
V_ss_ (mL/kg)	29.3 ± 4.08	32.7 ± 2.06	26.9 ± 2.04	59.3 ± 3.74	56.7 ± 6.66	53.5 ± 10.5
t_1/2_ (day)	0.96 ± 0.32	1.11 ± 0.097	1.19 ± 1.05	20.6 ± 1.66	19.2 ± 1.5	13.3 ± 4.42

AUC_0-∞_ = area under the concentration-time curve from time 0 to infinity; CL, clearance; C_max_ = maximum observed concentration; t_1/2_, _λz_ = terminal half-life; PK, toxicokinetic; V_ss_ = volume of distribution at steady state; SD, standard deviation; IV, intravenous; IL-15, interleukin 15; TaCk = targeted cytokine; PD-1, programmed cell death 1 receptor; RSV, respiratory syncytial virus.

The totality of findings in this study suggest the PD1/IL15 TaCk exhibits non-linear PK consistent with the expected target-mediated clearance, potentially attributed to expansion of target lymphocyte populations. However, the impact of ADAs on PK cannot be ruled out.

### PD1/IL15 TaCk demonstrated dose dependent PD-1^+^ lymphocyte expansion with selectivity over isotype control RSV/IL15 in cynomolgus monkeys

Single dose IV administration (dose range: 0.1–1.0 mg/kg) of PD1/IL15 TaCk exhibited PD-1-selective and expression-dependent lymphocyte expansion that resulted in substantial increases in PD-1^+^ lymphocytes and minimal increase of PD-1-low or negative lymphocyte populations (Figure 3). An initial decrease in peripheral PD-1^+^ lymphocyte counts was observed for 4–5 days in the single dose study, presumably due to margination and/or migration of these cells to the vessel wall and extravascular tissues, followed by an increase in several lymphocyte subsets, peaking on days 7–9 and returning toward pretreatment levels by 2–3 weeks post dose. Peak fold-expansion was dose-dependent with 9–87 fold (range) for PD-1^+^ CD8^+^ T cells, 9–19 fold for PD-1^+^ CD4^+^ T cells, 20–238-fold for CD4^+^ T_reg_, and 14–257 fold expansion for double negative (DN) T cells (Figure 3; Supplementary Table S4). The lymphocyte counts returned to at or near pre-study baseline values on day 21 for all animals. PD-1 low or negative cells such as NK cells, B cells, and granulocytes showed either minimal expansion or were not responsive to PD1/IL15 TaCk treatment. Among low PD-1 expressing lymphocytes, modest increases in naïve CD8^+^ and naïve CD4^+^ T cells were observed with peak fold expansion in the range of 2.5–10 fold (Supplementary Figure S2).

To assess whether memory subsets of CD8^+^ or CD4^+^ T cells responded more markedly to PD1/IL15 TaCk, CD8^+^ or CD4^+^ T cells were categorized as central memory (T_CM_), stem cell memory (T_SCM_), effector memory (T_EM_), and terminal effector (T_eff_) phenotype CD8^+^ or CD4^+^ cell populations according to the antibodies panel and gating strategies presented in Supplementary Table S5; Supplementary Figure S1. Dose-dependent cellular expansion was observed for all memory CD8^+^ or CD4^+^ T cell subsets (Supplementary Figure S2). The number of CD8^+^ T_eff_, T_EM_, and T_SCM_ cells were increased to a greater extent than of CD8^+^ T_CM_ cells. The expansion of CD8^+^ T_eff_, T_EM_ and T_SCM_ cells were in the range of 7–110 fold, 7–60 fold, and 9–61 fold, respectively, whereas the expansion of CD8^+^ T_CM_ was 6–29 fold across the tested dose levels (Supplementary Figure S2; Supplementary Table S4). Changes in the number of memory CD4^+^ T cell populations were generally lower compared to corresponding memory CD8^+^ T cell populations (Supplementary Figure S2). A steep, dose-dependent response from 0.3 mg/kg to 1 mg/kg was observed for all cell populations and their subsets, including DN T cells and T_regs_.

As expected, RSV/IL15 was completely inactive across the tested dose levels despite having greater exposure and prolonged PK. PD-1^+^ lymphocytes including all memory subsets were unresponsive to RSV/IL15 treatment. Following PD1/IL15 TaCk treatment, DN T cells, CD4^+^ T, CD8^+^ T cells in general, and specifically T_eff_, T_SCM_ and T_EM_ subsets, were highly expanded at their peak compared to the same cell types after RSV/IL15 treatment (Figure 3; Supplementary Figure S2). Overall, the PK/PD data from the single dose monkey study following PD1/IL15 TaCk and RSV/IL15 treatments suggested that differential PD-1 expression on antigen-specific lymphocytes enabled selective and avidity-driven targeting and confirmed the intended molecular mechanism of action of PD1/IL15 TaCk.

### PD1/IL15 TaCk demonstrated time-dependent and nonlinear PK in repeat-dose non-GLP and GLP cynomolgus monkey studies

**FIGURE 4:**
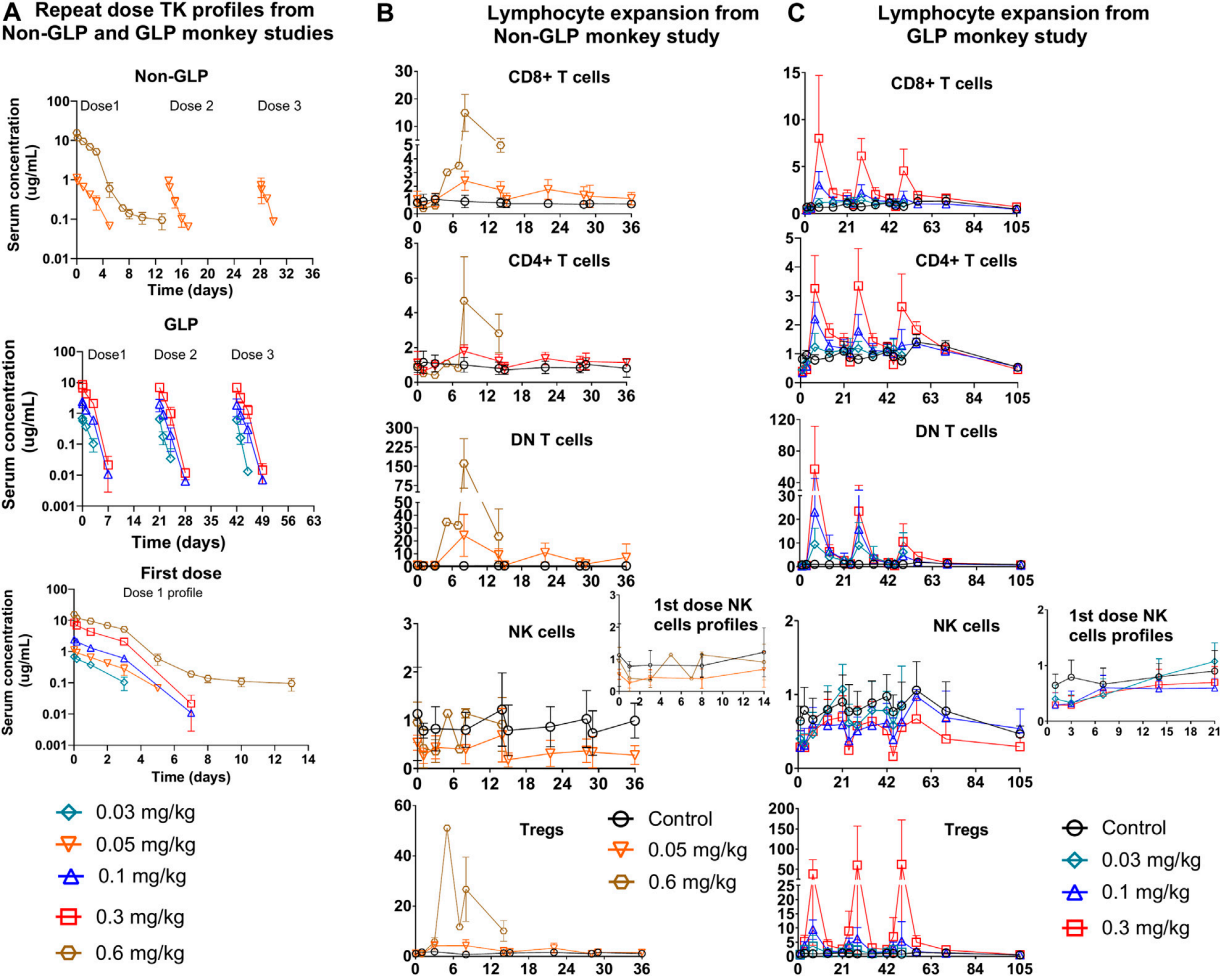
Repeat dose PKPD profiles from non-GLP and GLP cynomolgus monkey studies: **(A)** Repeat dose PK profiles of PD1/IL15 TaCk from non-GLP and GLP study. The PK/PD data for the 0.6 mg/kg dose group were only available for the first cycle due to unscheduled euthanasia on day 14. First dose cycle PK profiles are also presented **(B)** Non-GLP study: Dose-dependent lymphocytes (CD8^+^ T cells, CD4^+^ T cells, DN T cells [CD3^+^CD4–CD8–] and regulatory T cells) expansion following repeat IV administration (Q2W) in cynomolgus monkeys (n = 3/group). **(C)** GLP study: Dose-dependent lymphocyte (CD8^+^ T cells, CD4^+^ T cells, DN T cells (CD3^+^CD4^−^CD8^−^) and regulatory T cells) expansion following repeat IV administration (Q3W) in cynomolgus monkeys [In GLP study; n = 10 (5M/5F) for control, n = 6; (3F/3M) for 0.03 mg/kg, n = 10; (5F/5M) for 0.1 mg/kg, and n = 10; (5F/5M) for 0.3 mg/kg dose groups]. The data are represented as mean ± SD.

The TK of PD1/IL15 TaCk in the non-GLP study were characterized at the 0.05 and 0.6 mg/kg dose levels (N = 4/group), which were given Q2W for a total of three IV doses. The TK of PD1/IL15 TaCk for the 0.6 mg/kg dose group animals were available only for the first dose due to unscheduled euthanasia on Day 14 (Figure 4A). The increase in C_max_ was approximately dose proportional between 0.05 and 0.6 mg/kg doses and mean C_max_ for 0.05 mg/kg slightly decreased following repeated doses. Increases in mean exposure (AUC_0-∞, first dose_) were slightly more than dose proportional between 0.05 and 0.6 mg/kg of PD1/IL15 TaCk. Additionally, upon repeat dosing (post 2^nd^ dose of 0.05 mg/kg) a 65% decrease in exposure (AUC_0-∞_) was observed compared with the first dose (Table 3). Mean CL after the first dose ranged from 18.8 to 22.2 mL/day/kg (0.05–0.6 mg/kg) and increased to 66.8 mL/day/kg (0.05 mg/kg) upon repeat dosing; and mean t_1/2_ and V_ss_ after first dose ranged from 1.49 to 4.11 days and 41.1–45.3 mL/kg, respectively. ADAs were observed in all dosed animals (100% incidence), and animals with higher ADA titers (Supplementary Table S6) had lower exposure, indicating ADA-driven impact on exposure of PD1/IL15 TaCk. Overall, the PD1/IL15 TaCk demonstrated nonlinear PK behavior that was both dose- and time-dependent following IV administration.

**TABLE 3:** Summary of Mean (±SD) TK Parameters of PD1/IL15 TaCk following repeat dose IV administration to cynomolgus monkeys. Non-GLP study: Once every 2 weeks (Q2W) IV administration of 0.05 and 0.6 mg/kg PD/IL15 TaCk to male cynomolgus monkeys. GLP study: Once every 3-week (Q3W) IV administration of 0.03, 0.1 and 0.3 mg/kg in a 7-week repeat-dose cynomolgus monkeys (male and female) study with an 8-week recovery period.

TK parameter	Non-GLP study		GLP study		
	0.05 mg/kg, Q2W, IV (N = 4)	0.6 mg/kg^a^, Q2W, IV (N = 4)	0.03 mg/kg, Q3W (N = 6; 3M+3F)	0.1 mg/kg, Q3W (N = 10; 5M+5F)	0.3 mg/kg, Q3W (N = 10; 5M+5F)
C_max,_ first dose (µg/mL)	1.11 ± 0.0860	14.9^a^	0.701 ± 0.0914	2.41 ± 0.244	8.56 ± 1.28
C_max,_ second dose (µg/mL)	0.934 ± 0.0743	-	0.653 ± 0.120	2.11 ± 0.622	6.92 ± 1.09
C_max,_ third dose (µg/mL)	0.729 ± 0.381	-	0.612 ± 0.0867	1.85 ± 1.07	6.83 ± 1.55
AUC_0-∞,_ first dose (day•µg/mL)	2.30 ± 0.337	32.1^a^	1.17 ± 0.245	5.08 ± 0.444	16.5 ± 2.67
AUC_0-∞,_ second dose (day•µg/mL)	0.786 ± 0.198	-	0.824 ± 0.250	3.24 ± 0.808	11.5 ± 3.16
AUC_0-∞,_ third dose (day•µg/mL)	0.864^a^	-	0.654 ± 0.130	3.7 ± 1.04	12.0 ± 3.99
CL, first dose (mL/day/kg)	22.2 ± 3.82	18.8^a^	27.0 ± 7.63	19.8 ± 1.67	18.6 ± 3.01
CL, second dose (mL/day/kg)	66.8 ± 16.6	-	38.2 ± 11.6	32.4 ± 3.41	28.6 ± 10.4
CL, third dose (mL/day/kg)	58.6^a^	-	46.8 ± 9.26	28.9 ± 8.64	28.8 ± 14.0
V_ss_, first dose (mL/kg)	45.3 ± 10.1	41.1^a^	37.1 ± 5.50	40.0 ± 7.83	31.2 ± 8.67
t_1/2, λz_, first dose (day)	1.49 ± 0.422	4.11^a^	1.12 ± 0.311	1.41 ± 0.434	0.940 ± 0.321
AUC ratio (AUC _second_ _dose_/AUC_first_ _dose_)	0.341 ± 0.587	-	0.704 ± 1.01	0.637 ± 1.81	0.696 ± 1.18

AUC_0-∞,_ = area under the concentration-time curve from time 0 to infinity; CL, clearance; C_max_ = maximum observed concentration; t_1/2_, _λz_ = terminal half-life; V_ss_ = volume of distribution at steady state; Q2W = every 2 weeks, Q3W = every 3 weeks. SD, standard deviation; IV, intravenous; IL-15, interleukin 15; TaCk = targeted cytokine; PD-1, programmed cell death 1 receptor; PK, pharmacokinetics; a TK, parameters for the 0.6 mg/kg dose group were only available following the first dose due to unscheduled euthanasia on day 14.

^a^ Standard deviation (±SD) was not calculated due to insufficient data.

The TK of PD1/IL15 TaCk was further characterized in a 7-week repeat-dose, GLP toxicity study in monkeys. In this study, three dose levels (0.03, 0.1, and 0.3 mg/kg) of PD1/IL15 TaCk were administered Q3W for a total of 3 doses. Systemic exposure was confirmed in all animals and no sex differences were observed for PD1/IL15 TaCk exposure in monkeys (Figure 4A). The TK parameters were derived from individual serum concentration time data (Table 3). The C_max_ was dose-proportional after the first dose. There was a slight trend for decrease in C_max_ with repeated dosing; however, the ranges (mean ± SD) for C_max_ overlapped after the first, second, and third doses. After the first dose, AUC_0-∞_ showed a trend of slightly exceeding the dose proportionality However, upon repeat dosing, a substantial decrease (27%–44% decrease across tested dose levels) in exposure (AUC_0-∞_) was observed compared with the first dose. This decrease in systemic exposure (AUC_0-∞_) upon repeated dosing might be attributed to an increase in target mediated drug disposition (TMDD) (*i.e.,* as a result of increased target-cell population) in addition to potential impact of ADAs. The PD1/IL15 TaCk mean CL after the first dose ranged between 18.6 and 27.0 mL/day/kg and increased up to 28.8–46.8 mL/day/kg upon repeat dosing, which is approximately 2–5-fold higher than the CL for a typical IgG mAb in monkeys (<8 mL/day/kg) (Hötzel et al., 2012). Time and dose-dependent, non-linear PK behavior was observed for PD1/IL15 TaCk as indicated by a decrease in exposure and increase in CL upon second and third dose administration. No accumulation was observed following repeated administration as indicated by decreased AUC values, with an AUC ratio of 0.637- to 0.704-fold between the first and second doses (Table 3). In all baseline samples (including N = 10 control and N = 26 treatment groups), 3 out of 36 were ADA positive. The post-baseline ADAs to PD1/IL15 TaCk first appeared on Day 8. Twenty-four of twenty-six treated animals had ADAs to PD1/IL15 TaCk (92.3% ADA positive); however, only 4 of 24 ADA-positive animals had their exposure affected (undetectable levels; below the limit of quantification) upon the second or third dose. None of the animals in the vehicle control group were ADA-positive to PD1/IL15 TaCk. Supplementary Table S7 provided the range of titers for each group of animals. Overall, time and dose-dependent PK behavior was consistent for PD1/IL15 TaCk across non-GLP and GLP studies (Figure 4A).

### PD1/IL15 TaCk demonstrated dose-dependent PD-1^+^ lymphocyte expansion in repeat-dose cynomolgus monkey studies

Immune responses to PD1/IL15 TaCk were assessed in monkeys in non-GLP (Q2W dosing) and GLP (Q3W) repeat dose studies. In the non-GLP study, the peripheral cell expansion of PD1/IL15 TaCk for the 0.6 mg/kg dosed animals were only available following the first dose due to unscheduled euthanasia on Day 14. Dose-dependent changes in PD-1^+^ lymphocyte populations were observed following administration of PD1/IL15 TaCk in both studies (Figure 3). In general, reductions in absolute counts of peripheral T cell populations were observed at 24 h after administration of ≥0.03 mg/kg PD1/IL15 TaCk, followed by dose-dependent cell expansion typically peaking 7–8 days post-dose. Absolute counts of T cell populations generally returned to baseline (i.e., in the range as in the control group) by 14 days post-dose or prior to the next dose (day 21).

**FIGURE 5:**
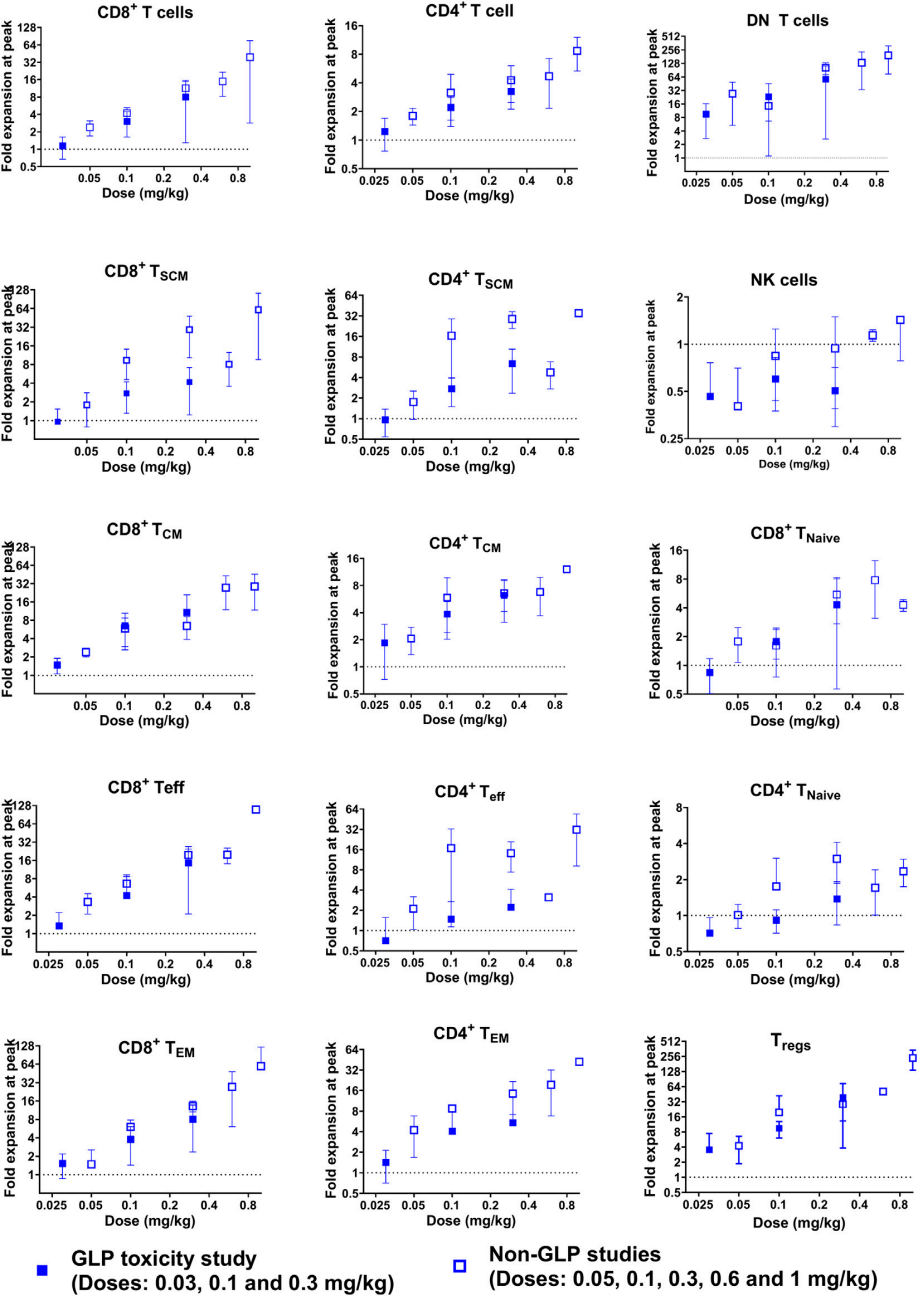
Expansion of CD8^+^ and CD4^+^ T cells, as well as their subsets [central memory (T_CM_), stem cell memory (T_SCM_), effector memory (T_EM_), and terminal effector (T_eff_) phenotype], DN T cells, and regulatory T cells (CD4^+^FoxP3^+^CD25^+^, T_regs_) was observed in a dose-dependent manner following single and repeat doses in non-GLP and GLP cynomolgus monkey studies. No NK cells (CD16^+^CD8a^+^) expansion was observed. The peak (mean ± SD) fold expansion after the first dose is presented here.

PD1/IL15 TaCk treatment resulted in dose-dependent expansion of lymphocytes with high PD-1 expression such as CD8^+^ T cells, CD4^+^ T cells and DN T cells (Figure 4). The peak fold of expansion (measured at day 7 and 8 post-dose in GLP study and non-GLP study, respectively) was dose-dependent with 1–15-fold for CD8^+^ T cells (2.7–23.4 fold for PD-1^+^ CD8^+^ T cells), 1.2–4.7-fold for CD4^+^ T cells (2.8–7.1 fold for PD-1^+^ CD4^+^ T cells, 3.4-51 for CD4^+^ T_reg_ cells), and 10–161 fold for DN T cells at the dose range of 0.03–0.6 mg/kg after the first dose in the non-GLP and GLP studies (Supplementary Table S4). The lymphocyte counts returned to pre-study baseline values on Day 70 (no measurement between day 56 and 70) for all recovery animals from 0.1 to 0.3 mg/kg of dose in the GLP study (Figure 4). In general, cell populations that are PD-1 low or negative showed minimal expansion or were non-responsive to PD1/IL15 TaCk. Within the CD8^+^ T cell and CD4^+^ T cell compartments, dose-dependent cell expansions were observed in T_CM_, T_SCM_, T_EM_, and T_eff_ subsets (Figure 5). The peak fold expansion of CD8^+^ T_CM_, T_SCM_, T_eff_ and T_EM_ cells after the first dose were in the range of 1.5–28 fold, 1–8 fold, 1.3–20 fold, 1.3–20 fold and 1.5–27 fold, respectively, for the tested dose range of 0.03–0.6 mg/kg across both studies (Figure 5; Supplementary Table S4). No or minimal expansion was observed at 0.03 mg/kg for all cell types with the exception of a 10-fold expansion observed for DN T cells, and a 3.5-fold expansion of CD4^+^ T_regs_. Substantial expansion of memory CD4^+^ T cells populations were observed; however, it was generally lower compared to corresponding memory CD8^+^ T cell populations (Figure 5). Furthermore, an attenuation of proliferation measured as a decreased peak of expansion in PB was observed following the second or third dose for highly proliferative cells (e.g., CD8^+^ T cells, CD4^+^ T cells, DN T cells) at the doses of 0.1 and 0.3 mg/kg Q3W in the GLP study (Figure 4; Supplementary Table S8). The frequency of Ki-67 was also measured in these studies. Administration of ≥0.03 mg/kg PD1/IL15 TaCk resulted in dose-dependent increases in proliferating T cell populations as measured by %Ki-67 consistent with absolute cell numbers (Supplementary Figure S3). Frequencies of Ki-67 + T cell populations generally peaked 3–7 days post-dose and returned to baseline and control ranges 14 days post-dose or prior to next dose (day 21). In the GLP study, administration of ≥0.03 mg/kg PD1/IL15 TaCk resulted in dose-dependent increases in the frequency of Ki-67+ in PD-1^+^ CD8^+^, CD4^+^, DN T cells and T_regs_ (Supplementary Figure S3). Overall, PD1/IL15 TaCk showed dose-dependent and selective proliferation and expansion of PD-1^+^ lymphocytes (CD8^+^, CD4^+^ T cells and memory subsets, DN T cells and T_regs_) in monkeys across the studies. As anticipated, either no effects or minimal effects of PD1/IL15 TaCk were observed on lymphocytes with low PD-1 expression such as NK cells (CD16^+^CD8a^+^), naïve CD8^+^ and CD4^+^ T cells. However, a modest increase of other less abundant NK cell subtypes (CD56^+^CD16^−^, CD56^−^CD16^+^, CD16^+^CD8a^-^) were observed and likely attributed to PD-1 expression on these subtypes (Supplementary Figure S4).

### FIH dose and dosing regimen selection

The FIH dose of PD1/IL15 TaCk was determined using a MABEL approach due to the drug’s immune agonistic properties (Saber et al., 2016; Saber et al., 2017). The FIH dose was derived using *in vitro* CD4^+^ T_EM_ cell (CD45RA–CCR7–CD28+/–CD95+) proliferation (percent of cells with CFSE staining) in stimulated human PBMCs, the most sensitive *in vitro* assay using PD1/IL15 TaCk. A dose of 0.003 mg/kg IV is proposed as the FIH dose for PD1/IL15 TaCk. This was based on EC_50_ (0.070 μg/mL; geometric mean of 8 donors) of PD1/IL15 TaCk for CD4^+^ T_EM_ cell proliferation (Table 1) and physiological plasma volume of 42.9 mL/kg in humans (Davies and Morris, 1993).The predicted C_max_ of PD1/IL15 TaCk after IV administration of 0.003 mg/kg in humans is not expected to exceed this EC_50_ level.

Based on target cell expansion (CD8^+^ or CD4^+^ effector memory T cells) in tumor and peripheral blood (data not shown), 0.01 mg/kg of PD1/IL15 TaCk was identified as mPAD in a humanized (hCD34+ engrafted in NSG) mouse model. The mPAD in the mouse model is predicted to be exposure equivalent to a human dose of 0.0077 mg/kg using predicted human CL of 11.6 mL/day/kg (scaled from monkey CL of 18.6 mL/day/kg utilizing an exponent of 0.85 (Deng et al., 2011)). This mouse mPAD equivalent human dose is 2.6-fold higher than the proposed PD1/IL15 TaCk FIH dose of 0.003 mg/kg. Therefore, the proposed starting dose of 0.003 mg/kg in patients is expected to be associated with minimal pharmacological activity.

The proposed dosing frequency of PD1/IL15 TaCk in humans is Q3W and is supported by the 7-week GLP toxicity study in monkeys where peak peripheral PD response (target-cell expansion such as CD8^+^ and CD4^+^ T cells and their effector memory subsets) was achieved a week after dosing and peripheral target cell counts were declining toward their baseline by the end of 3 weeks. Furthermore, cytokines and chemokines indicative of PD activity peaked between 24–72 h following dosing and returned to baseline within 21 days of dosing (data not shown). Therefore, an initial dosing frequency of Q3W is considered appropriate in the Phase I dose escalation study of PD1/IL15 TaCk with the dose-limiting toxicity observation period encompassing the first cycle of study treatment.

## Discussion

Immunotherapy has improved treatment outcomes for cancer patients, and ongoing efforts include a focus on harnessing the therapeutic potential of cytokines such as IL-2 and IL-15 in various cancer indications. While IL-2 is an approved cancer immunotherapy, IL-15 has not yet established clinical benefit. To optimize IL-15 based therapies, we engineered PD1/IL15, a recombinant targeted cytokine that selectively delivers IL-15 to lymphocytes that express PD-1. We characterized the PK and PD of PD1/IL15 TaCk in single and repeat-dose cynomolgus monkeys to establish PK/PD relationships and inform future clinical studies. Excitingly, we confirmed the selective, avidity-driven targeting of PD1/IL15 TaCk on PD-1^+^ lymphocytes by using an isotype control RSV/IL15 that lacked PD-1 targeting, which supports the hypotheses driving our molecular design and mechanism of action.

After a single IV dose, PD1/IL15 TaCk exhibited a multiphasic non-linear PK profile with CL higher than a typical target-independent IgG CL in monkeys (Hötzel et al., 2012). In contrast, the isotype control RSV/IL15 showed a linear and typical IgG-like PK in monkeys (Hötzel et al., 2012; Leipold et al., 2018). The higher than normal IgG CL of PD1/IL15 TaCk compared to RSV/IL15 could be attributed to both the enhanced TMDD expected from increased lymphocyte expansion in response to PD1/IL15 TaCk as well as the effect of ADA occurrence. In repeat dose studies, a target-enhanced TMDD effect (*i.e.,* dynamic TMDD due to target cell expansion) resulted in time-dependent increase in CL and substantial decrease in AUC upon repeated dosing (compared to 1^st^ dose). Similar to the single dose study, the CL values in repeat dose studies were approximately 2–5-fold higher than the CL for a typical IgG mAb in cynomolgus monkeys (Hötzel et al., 2012). Published data also indicates that IL-15 or IL-2 can induce a “cytokine sink” by promoting the proliferation and expansion of target lymphocytes. This can trigger a corresponding increase in the consumption/clearance of IL-15, ultimately leading to “dynamic TMDD”(Hua et al., 2019). The dynamic TMDD observed for PD1/IL15 TaCk is similar to the PK/PD interaction seen with therapeutics that enhance receptor expansion and thereby increase TMDD-driven clearance resulting in time-dependent PK (Hua et al., 2019). The time-dependent PK behavior of PD1/IL15 TaCk observed in our study has also been reported for other IL-2 and IL-15 based therapeutics (Sneller et al., 2011; Conlon et al., 2019b; Lu et al., 2023). Continuous infusion of rhIL-15 in monkeys and in humans resulted in the expected steady state level of serum IL-15 initially, then decreased ∼4–5 fold over time (Sneller et al., 2011; Conlon et al., 2019b). Sequential SC administration of the same doses of the super-agonist hetIL-15 resulted in decreasing C_max_ and C_min_ upon repeated injections (Bergamaschi et al., 2018). In addition, PK modeling of cergutuzomab amunaleukin (CEA-IL-2v), an IL-2-based carcinoembryonic antigen-targeted cytokine, revealed expansion of a drug-induced peripheral sink leading to increased clearance (Ribba et al., 2018). Overall, time-dependent, non-linear PK behavior was observed for PD1/IL15 TaCk as indicated by a decrease in exposure and increase in CL upon second and third dose administration. In addition to the observed dynamic TMDD, an impact of ADAs on PK of PD1/IL15 TaCk cannot be excluded. Immunogenicity incidence in the GLP study was high (92.3%, 24 of 26 PD1/IL15-treated animals were ADA positive). However, the impact of ADA on exposure appeared to be minimal since only 4 of 24 ADA-positive animals had their exposure affected. Furthermore, ADA incidence may not be predictive of what will be observed in humans since the translatability of ADA incidence from cynomolgus monkey to human for this molecule is unknown.

Various protein engineering strategies are under investigation to increase the duration of cytokine exposure in humans, extending the half-life (t_1/2_) from hours to days or even longer. However, the success of engineered half-life extended IL-15 agonists has been limited thus far-systemic elimination t_1/2_ of IL-15 variants have ranged from only several hours to 1 day (Waldmann et al., 2011; Rhode et al., 2016; Miyazaki et al., 2021). One exception to this trend is efbalropendekin alfa, an engineered IL-15/IL-15Rα-Fc fusion protein that exhibited a t_1/2_ of approximately 2.5–4.5 days in both single- and repeat-dose monkey studies (Lu et al., 2023). PD1/IL15 TaCk continues that trend, with substantially prolonged PK (t_1/2_–1.0–4.1 days) compared to other engineered IL-15 therapeutics of various formats. This is a result of both the reduced IL-15 potency of PD1/IL15 TaCk by several hundred-fold on non-activated cells coupled with Fc engineering to increase affinity to FcRn at pH 6 (Waldmann et al., 2011; Rhode et al., 2016; Miyazaki et al., 2021), which together reduce TMDD and enhance FcRn mediated recycling of the TaCk.

PD1/IL15 TaCk demonstrated a dose-dependent expansion of PD-1^+^ lymphocytes, whereas minimal or no expansion of lymphocytes with low PD-1 expression, which was confirmed through an isotype control RSV/IL15 that lacked PD-1 targeting showing only a maximum expansion of 2.1-fold at 1 mg/kg despite having greater exposure and prolonged PK. Analysis of the PD effects of PD1/IL15 TaCk on multiple immune cell subtypes revealed the substantial expansion of CD8^+^ and CD4^+^ T_CM_, T_SCM_, T_EM_, and T_eff_ T cell populations as well as T_regs_ after single- and repeat-dose administration. In general, PD-1^+^ lymphocytes, including all memory subsets, were unresponsive towards RSV/IL15 treatment. These results clearly indicate PD1/IL15 TaCk treatment resulted in selective and avidity-driven targeting, and dose-dependent expansion of PD-1^+^ lymphocytes, validating the molecule design and mechanism of action. We observed negligible or minimal effects of PD1/IL15 TaCk on lymphocytes with low PD-1 expression such as NK cells. This PD-1-selective, avidity-driven peripheral expansion of immune effector cells with potential antitumor activity may provide therapeutic benefit and supports future clinical studies with PD1/IL15 TaCk as a monotherapy or in combination with anti-PD-1 or anti-PD-L1 checkpoint inhibitors in patients with advanced solid tumors. However, the dose-dependent expansion of peripheral T_regs_ in response to PD1/IL15 TaCk treatment and their potential effect on antitumor immunity is unknown and needs further investigation.

Additionally, the therapeutic benefit of PD1/IL15 TaCk will need to be carefully considered in light of the possible therapeutic window and overlap of pharmacodynamic activity, due to PD1+ immune cell expansion, with toxicity, due to lymphocyte infiltration and associated injury, into normal tissues. Notably, high doses (0.6 mg/kg) of PD1/IL15 TaCk resulted in intolerability and early euthanasia in cynomolgus monkeys which was attributed to widespread and rapid T cell expansion, activation, and infiltration into tissues, especially in the gastrointestinal tract, kidneys, and heart, resulting in injury to these organs.

It is worth noting that memory phenotype (CD8^+^ and CD4^+^ T cells memory subsets) populations were markedly expanded upon PD1/IL15 TaCk treatment. Interestingly, the peak fold expansion of CD8^+^ and CD4^+^ T cell memory subtypes following PD1/IL15 TaCk treatment are substantially higher than peak fold expansion of corresponding memory T cell populations in response to rhIL-15 (Lugli et al., 2010), and nemvaleukin (Lopes et al., 2021), (a novel engineered IL-2) in monkeys. PD1/IL15 TaCk not only enhanced the magnitude of selective target cell expansion, but also improved the duration of cell expansion compared to other engineered IL-15 therapeutics in cynomolgus monkeys or rhesus macaques (Waldmann et al., 2011; Rhode et al., 2016; Miyazaki et al., 2021), potentially enabling a less frequent dosing option for patients. Consistent with increases in target cell population, PD1/IL15 TaCk demonstrated a dose-dependent increase in Ki-67+ (%Ki-67) lymphocytes, with peak response achieved after a week of dosing. The modest decrease in the magnitude of peak expansion of circulating DN T cells, CD4^+^ T cells and CD8^+^ T cells as well as %Ki-67+ (Supplementary Figure S3) following the second or third dose of PD1/IL15 TaCk could potentially be driven by lower exposures upon repeat dosing. This in turn is assumed to be contributed by dynamic TMDD effect in addition to potential impact of ADAs on exposure. Overall, the PK/PD data of PD1/IL15 TaCk in monkeys suggests sustained PK, durable PD response (target cell expansion), and less frequent dosing option for patients.

Over the past decade, both the EMA and the FDA have endorsed MABEL-based recommendation of FIH dose for immune activating agents such as checkpoint inhibitors, T cell bispecific antibodies and cytokine fusions (FDA, 2005; EMEA, 2007). Saber et al (Saber et al., 2016), in a FDA publication compared various MABEL-based approaches for immuno-oncology drugs employed by several sponsors, which targeted 20%–80% of EC or receptor occupancy (RO) from either *in vitro* or *in vivo* studies. The MABEL approach is comprehensive, typically considers totality of data including *in vitro/ex vivo* studies, preclinical pharmacology, toxicology investigations, *in vivo* models, and pharmacodynamic modeling, and aims to recommend a safe starting dose for human trials. We based our MABEL approach on the concentration where PD1/IL15 TaCk showed 50% of its maximum activity (EC_50_) in the most sensitive *in vitro* assay and converted that concentration into a human equivalent dose that would result in similar C_max_ concentration. The CD4^+^ T_EM_ cell (CD45RA-CCR7-CD28+/-CD95^+^) was the most sensitive among pharmacologically relevant cell populations and EC_50_ of *in vitro* proliferation of this cell subset by PD1/IL15 TaCk translates to a proposed starting dose of 0.003 mg/kg in patients (Table 1; Supplementary Figure S6). This proposed dose is consistent with starting doses of other clinically evaluated IL-15 analogs, such as rhIL-15 (Conlon et al., 2015; Miller et al., 2018; Conlon et al., 2019b), and IL-15 superagonist ALT-803 (Margolin et al., 2018; Romee et al., 2018), that have been shown to be well tolerated at their respective clinical starting doses. Furthermore, the mPAD (0.01 mg/kg) in hCD34^+^ NSG-engrafted mouse model (data not shown) is predicted to be exposure equivalent to a human dose of 0.0077 mg/kg, which is 2.6-fold higher than the currently proposed PD1/IL15 TaCk starting dose of 0.003 mg/kg. Therefore, the proposed starting dose of 0.003 mg/kg is predicted to be a safe starting dose for FIH studies and associated with minimal pharmacological activity.

## Conclusion

In summary, PD1/IL15 TaCk exhibited dose- and time-dependent, multiphasic, nonlinear PK behavior in monkeys. The non-linearity is predominantly attributed to dynamic TMDD, in addition to ADA impact. The TaCk demonstrated dose-dependent and PD-1^+^-selective lymphocyte expansion in monkeys following single- and repeat dose administrations. The expansion of PD1^+^ effector lymphocytes encourages the potential combination with lymphocyte-targeted cancer immunotherapies; on the other hand, the expansion of PD-1^+^ T_regs_ may counteract this effect and need further investigation of Treg mediated effects (Kumagai et al., 2020). A conservative MABEL-based approach coupled with *in vivo* PK/PD in monkeys led to a recommended FIH clinical dose of 0.003 mg/kg administered Q3W. The combination of relevant nonclinical data and understanding complex biological relationships (PK/PD) allowed us to guide the selection of starting clinical dose and dosing regimens. Overall, this work provides a translational research framework to study emerging cytokine modalities and inform FIH clinical trials.

## Data Availability

The original contributions presented in the study are included in the article/Supplementary Material, further inquiries can be directed to the corresponding authors.
