# Supplementary material for: Translational PK/PD and the first-in-human dose selection of a PD1/IL15: an engineered recombinant targeted cytokine for cancer immunotherapy
Source: Front Pharmacol. 2024 Jun 3;15:1380000. doi: 10.3389/fphar.2024.1380000 (PMC11181026; doi:10.3389/fphar.2024.1380000)
Supplement: Supplementary file 1 [file DataSheet1.docx]

**Supplementary Materials**

**Translational PK/PD and the First-in-Human Dose Selection** **of a PD1/IL15: An Engineered Recombinant Targeted Cytokine for Cancer Immunotherapy**

Rajbharan Yadav^1‡*^, Suzanne Schubbert^2‡^, Patrick G. Holder^1^, Eugene Y. Chiang^1^, Nargess Kiabi^2^, Liz Bogaert^2^, Irene Leung^2^, Rumana Rashid^2^, Kendra N. Avery^2^, Christine Bonzon^2^, John R. Desjarlais^2^, Shomyseh Sanjabi^1^, Amy Sharma^1^, Michelle Lepherd^1^, Amy Shelton^1^, Pam Chan^1^, Yanqiu Liu^1^, Louis Joslyn^1^, Iraj Hosseini^1^, Eric G. Stefanich^1^, Amrita V. Kamath^1^, Matthew J. Bernett^2*^, Vittal Shivva^1*^

^1^Genentech, Inc., South San Francisco, CA, USA

^2^Xencor, Inc. Pasadena, CA, USA

^‡^Authors contributed equally

^*^Corresponding authors

**Supplemental Methods**

**Ethics statement**

The authors confirm that they have obtained appropriate institutional review board approval for all animal experimental investigations at both Altasciences (Seattle, WA, USA) and Charles River Laboratories (Reno, NV, USA). All procedures were approved by the Institutional Animal Care and Use Committee at Altasciences and at Charles River Laboratories and were performed in compliance with the Animal Welfare Act, the Guide for the Care and Use of Laboratory Animals, and the Office of Laboratory Animal welfare. PK and PD (absolute counts of lymphocytes) of the PD1/IL15 TaCk after single- and repeat-dose administrations were evaluated in two non-good laboratory practice (non-GLP) studies (conducted one each at Altasciences and CRL), and a GLP study (conducted at CRL) in monkeys. RSV/IL15 was investigated as a control group in the single dose non-GLP study conducted at Altasciences. Healthy volunteer PBMCs donor eligibility criteria were met as per FDA guidance document titled "Guidance for Industry: Eligibility Determination for Donors of Human Cells, Tissues, and Cellular and Tissue-Based Products (HCT/Ps)". Furthermore, donor eligibility was confirmed based on these criteria specified in 21 CFR Part 1271 and in accordance with current approved SOPs for each respective collection. All 8 donors used in this study had passed the screening/testing to be considered as part of our normal healthy donor pool.

**Dose ranging PK/PD (Non-GLP) of PD1/IL15 TaCk and RSV/IL15 in cynomolgus monkeys**

Eighteen male monkeys were randomly assigned to six groups (n = 3 animals/group) and were given a single IV dose (0.1, 0.3, or 1 mg/kg) of either PD1/IL15 or RSV/IL15. Blood samples (0.5 mL) for PK, PD (peripheral blood immunophenotyping) and anti-drug antibodies (ADA) were collected from each animal via the femoral vein on day 0 (pre-dose, 5 minutes, 6 and 16 hours post- dose), and 1, 2, 3, 4, 5, 6, 7, 9, 11, 14, 16, 18, 21, 24, and 28 days post-dose.

**Non-GLP repeat-dose toxicokinetics (TK) of PD1/IL15 TaCk in cynomolgus monkeys**

Naïve male monkeys (n=4/dose group) were given slow IV bolus administration of 0.05 mg/kg and 0.6 mg/kg of PD1/IL15 TaCk, once every two weeks (Q2W) for a total of three dose cycles (on days 0, 14, and 28). Blood samples for PK (0.8 mL), PD (1 mL) and ADA (0.5 mL) analyses were collected from each animal via saphenous venipuncture. Serum PK of PD1/IL15 was quantified pre-dose, 15 minutes, 4 and 8 hours post-first, second and third dose, and 1, 2, 3, 5, 8, 10, 14, 15, 16, 17, 19, 22, 24, 28, 29, 30, 31 and 36 days. Serum for ADA analysis was collected at pre-dose (two weeks prior to dosing), and at days 8, 14, 22, 28, and 36 post-dosing. Blood samples for PD (immunophenotyping) were collected at designated time points (pre-dose (Week-1), and on day 1, 3, 8, 14, 15, 22, 28, 29 and 36 post-dosing) for flow cytometric evaluation of various lymphocyte populations.

**7-week repeat-dose GLP toxicity study of PD1/IL15 TaCk administered via IV infusion in cynomolgus monkeys with an 8-week recovery period**

Naïve male and female monkeys (total 26 animals) were given slow IV bolus administration of 0.03 mg/kg (n=6; 3F and 3M), 0.1 mg/kg (n=10; 5F and 5M) and 0.3 mg/kg (n=10; 5F and 5M) of PD1/IL15 TaCk, once every three week (Q3W) for a total of three dose cycles (on days 0, 21, and 42). Blood samples for PK (0.5 mL), PD (1.8 mL) and ADA (0.65 mL) analyses were collected from each animal via saphenous venipuncture. Serum PK of PD1/IL15 TaCk was quantified pre-dose, 15 minutes, and 8 hours post-first, and 15 minutes post-second and third dose, and on days 1, 3, 7, 14, 21, 22, 24, 28, 35, 42, 43, 45, 49, and 56. ADA samples were collected pre-dose (day 0) and at days 7, 14, 21, 28, 35, 42 , 49, 56, 70 and 105 post-dosing. Blood samples for flow cytometric evaluation of various lymphocyte populations were collected at designated time points (pre-dose Week-1, day 0), and on days 1, 3, 7, 14, 21, 22, 24, 28, 35, 42, 49, 56, 70 and 105. Additional PK, PD and ADA samples from recovery animals (n = 4/group; 2F/2M, out of a total of 10 animals each in 0.1 and 0.3 mg/kg dose groups) were collected for 15 weeks on days 70 and 105 after the third dose of PD1/IL15 TaCk.

**Anesthesia and Euthanasia procedure in monkey studies:**

Animals were fasted prior to anesthesia. The animals were given Buprenorphine SR (0.20 mg/kg) subcutaneously (SC) and Meloxicam SR (0.6 mg/kg) SC one time on the day of surgery prior to the biopsy procedure. Animals were pre-anesthetized with Ketamine HCL (10-15 mg/kg) intramuscularly (IM). Dexdomitor (0.06 mg/kg) IM was administered for additional anesthesia. Local analgesic (i.e., Bupivicaine 0.25%, 0.20 mL) were administered at each site. The drug, dose, route and site of administration was documented in the surgical records.

Animals surviving until scheduled euthanasia will have a terminal body weight recorded, blood or urine samples collected as specified in the protocol, and were euthanized by intravenous injection of a commercially available veterinary euthanasia solution containing a mixture of phenytoin (50 mg/ml) and pentobarbital (390 mg/ml), followed by exsanguination. When possible, the animals were euthanized rotating across dose groups such that similar numbers of animals from each group, including controls, were necropsied throughout the day. Animals were fasted before their scheduled necropsy.

***PK Assay*:** The serum samples from single dose monkey study were analyzed for PD1/IL15 TaCk and RSV/IL15 at Xencor (Monorovia, CA, USA) using a Sandwich DELFIA® TRF method. Goat anti-human IL-15Rα antibody (R&D Systems, Minneapolis, MN, USA) and recombinant RSV-F (Sino Biologicals; Chesterbrook, PA, USA) were used as capture, while Bio-XENP15595 (Xencor, Lot No. PT20190614) and mouse anti-human/primate IL-15 biotinylated antibody (Catalog No. BAM247) were used as primary detection reagents for PD1/IL15 TaCk and RSV/IL15 respectively. DELFIA^®^ Europium-labeled streptavidin (PerkinElmer, Catalog No. 1244-360) were used as secondary detection reagents for both molecules. The lower limit of quantification (LLOQ) was 32.8 ng/mL and 97.7 ng/mL for PD1/IL15 TaCk and RSV/IL15, respectively. Serum samples from repeat dose-non-GLP monkey study were analyzed for PD1/IL15 using a quantitative enzyme-linked immunosorbent assay (ELISA). Briefly, this ELISA uses a sheep anti-human immunoglobulin (IgG) heavy and light chain antibody as the capturing reagent and goat anti-human IgG heavy and light chain conjugated to horseradish peroxidase (HRP) as the detecting reagent. The LLOQ was 62.5 ng/mL. An ELISA method was developed and validated to quantify PD1/IL15 in monkey serum samples from GLP tox study. Briefly, goat anti-human IL-15Rα antibody was used as capture, while biotinylated PD-1 and HRP labelled streptavidin were used as primary and secondary detection reagents. The LLOQ in this assay was 5 ng/mL.

***ADA Assay****:*

A direct coat ELISA was developed to assess ADA against PD1/IL15 TaCk in the monkey serum samples from single dose and repeat dose non-GLP study. The assay utilized mouse anti-human-Fc and PD1/IL15 TaCk as a capture for ADA samples from single dose and repeat dose study, respectively. The goat anti-monkey IgG (heavy and light chain) conjugated to HRP was used to detect ADA against PD1/IL15 TaCk. The sensitivity of the assay was 1.7 titer units (log_10_50), with a minimum sample dilution of 1/50. Animals with an ADA titer of <1.7 were considered as ADA negative. ADA samples from RSV/IL15-treated animals were not analyzed. A bridging ELISA method was developed and validated to detect PD1/IL15 in monkey serum samples from the GLP toxicity study. The assay utilized a biotinylated PD1/IL15 TaCk and a digoxigenin-labeled PD1/IL15 TaCk to capture and detect ADAs. Using an affinity-purified, mouse anti-human IL-15 antibody as positive control, the relative sensitivity for screening assay was determined to be 384 ng/mL. The relative Sensitivity is calculated using following formula: (Concentration above cut point + {[(mean signal above cut point – cut point) * (concentration below cut point – concentration above cut point)] / (mean signal above cut point – mean signal below cut point)}. Using the same positive control at 1,000 ng/mL, the drug tolerance levels were determined to be 1 µg/mL.

***Peripheral Blood Immunophenotyping****:* Flow cytometry method was used to measure various lymphocyte and their subtypes. The cellular antigens and cell populations identified were quantified, using specific antibodies panel and gating strategies (Supplement Table S4 and Figure S1). BD TruCount™ tubes were used in combination with CD45/Side Scatter gating for the real time quantification of absolute cell counts, per Testing Facility Standard Operating Procedure. The lymphocyte subset percentages were taken from the BD TruCount™ data and applied to the total number of lymphocytes. For flow cytometry analysis, peripheral blood samples were treated with RBC Lysis Buffer (eBiosciences) for 15-20 min at room temperature (RT), washed and centrifuged prior to resuspension of cell pellet in Stain Buffer (FBS) (BD Biosciences) and centrifugation. Cells were then incubated in Blocking Buffer Solution containing Human Fc Block (BD Biosciences) and mouse serum (Jackson ImmunoResearch) for 10-15 min at RT protected from light, followed by addition of antibody cocktail and incubation for another 15-20 min at RT protected from light. Cells were then fixed in solution consisting of 0.2% paraformaldehyde (EMS) in PBS. Sample acquisition was performed using the LSR Fortessa X-20 instrument (BD), and flow cytometry data analyzed using DIVA® software.

***PK data analysis****:* Non-compartmental analysis (NCA) method was used to estimate the PK parameters from serum concentration-time profiles obtained in non-GLP and GLP studies, using Phoenix™ WinNonlin®, Version 6.4 software (WinNonlin; Certara, Inc., USA, NJ). Each animal was analyzed separately and results for each dose group were summarized as mean ± standard deviation (SD). AUC was calculated using the log-linear trapezoidal rule. Nominal sample collection times and nominal dose solution concentrations were used in PK data analysis. For the single-dose studies in monkeys, maximum concentration post dose administration (C_max_), area under the serum concentration-time curve extrapolated to infinity (AUC_0-∞_ ), systemic clearance (CL), and volume of distribution at steady state (V_ss_) were calculated. For the repeat-dose cynomolgus study, C_max_ after first drug administration (PK day 0), second (PK day 14 or 21) and third drug administration (PK day 28 or 42), AUC in the first dosing interval (AUC_0-∞, 1st dose_), second dosing interval (AUC_0-∞, 2nd dose_), and third dosing interval (AUC_0-∞, 3rd dose_) were obtained. Accumulation ratios (AUC_0-∞, 2nd dose_ /_AUC0-∞, 1st dose_) for male and females were calculated from the GLP study.

**Supplemental Tables**

**Table S1*.*** *In vitro* potency of PD1/IL15 TaCk on stimulated hPBMC (Geometric mean from 8 healthy human donors)

| **ECx (ng/mL)** | ***In vitro* potency on stimulated hPBMC (Geometric mean from 8 healthy human donors)** | | | |
| --- | --- | --- | --- | --- |
|  | **CD4^+^ T cell** | **CD4^+^ Effector memory** | **CD8^+^ T cell** | **CD8^+^ Effector memory** |
| EC_10_ | 19.8 | 13.9 | 28.5 | 22.3 |
| EC_20_ | 37.7 | 25.2 | 58.9 | 43.0 |
| EC_30_ | 57.9 | 37.4 | 95.3 | 66.43 |
| EC_40_ | 82.4 | 51.8 | 141.4 | 95.0 |
| EC_50_ | 113.8 | 69.8 | 203.1 | 131.8 |
| EC_60_ | 157.2 | 94.0 | 291.7 | 183.0 |
| EC_70_ | 223.5 | 130.1 | 432.8 | 261.5 |
| EC_80_ | 343.3 | 193.3 | 700.5 | 404.4 |
| EC_90_ | 655.1 | 350.8 | 1445.3 | 779.2 |

IL-15 = interleukin 15; TaCk = targeted cytokine; PD-1 = programmed cell death 1 receptor; hPBMC = human peripheral blood mononuclear cells

**Table S3:** Parameter variability, precision, and goodness of fit of curves after using a 4 model parameters model for ECs values calculations.

| **Sigmoidal dose-response (variable slope, no Log transform)** | **Donor ID** | | | | | | | |
| --- | --- | --- | --- | --- | --- | --- | --- | --- |
|  | **142802** | **152994** | **160124** | **D205004** | **D205833** | **D208979** | **D326828** | **D329100** |
| **Best-fit values** |  |  |  |  |  |  |  |  |
| Bottom | 24.8 | 8.679 | 38.8 | 14.26 | 35.76 | 30.68 | 48.49 | 20.07 |
| Top | 89.18 | 79.43 | 76.08 | 77.46 | 88.84 | 83.69 | 74.94 | 75.2 |
| EC_50_ | 49.76 | 287.1 | 171.4 | 78.65 | 127.1 | 302.3 | 123.4 | 99.77 |
| HillSlope | 1.177 | 1.169 | 1.473 | 1.48 | 1.062 | 0.9843 | 1.423 | 1.334 |
| LogEC_50_ | 1.697 | 2.458 | 2.234 | 1.896 | 2.104 | 2.48 | 2.091 | 1.999 |
| **Standard Error** |  |  |  |  |  |  |  |  |
| Bottom | 1.567 | 1.662 | 2.438 | 1.934 | 0.9826 | 3.608 | 1.105 | 1.185 |
| Top | 0.858 | 1.614 | 2.081 | 1.323 | 0.7259 | 3.552 | 0.8602 | 0.8535 |
| EC_50_ | 4.617 | 34.47 | 49.63 | 9.374 | 10.8 | 111.3 | 21.84 | 8.938 |
| HillSlope | 0.1126 | 0.1471 | 0.5399 | 0.2298 | 0.08645 | 0.3276 | 0.3166 | 0.1417 |
| **95% CI (profile likelihood)** |  |  |  |  |  |  |  |  |
| Bottom | 20.87 to 28.23 | 4.551 to 12.41 | 31.71 to 44.08 | 9.373 to 18.53 | 33.29 to 37.97 | 20.87 to 37.90 | 45.16 to 50.98 | 16.94 to 22.82 |
| Top | 87.19 to 91.26 | 75.61 to 83.81 | 71.24 to 82.31 | 74.22 to 80.95 | 87.15 to 90.64 | 76.01 to 95.85 | 72.93 to 77.23 | 73.20 to 77.31 |
| EC_50_ | 39.81 to 61.71 | 216.6 to 386.0 | 86.82 to 377.8 | 59.79 to 105.3 | 103.8 to 155.1 | 126.5 to 862.5 | 78.75 to 188.5 | 80.25 to 123.0 |
| HillSlope | 0.9470 to 1.474 | NA | 0.5894 | NA | 0.8741 to 1.298 | 0.4689 to 2.289 | NA | NA |
| LogEC_50_ | 1.600 to 1.790 | 2.336 to 2.587 | 1.939 to 2.577 | 1.777 to 2.022 | 2.016 to 2.191 | 2.102 to 2.936 | 1.896 to 2.275 | 1.904 to 2.090 |
| **Goodness of Fit** |  |  |  |  |  |  |  |  |
| Degrees of Freedom | 7 | 7 | 7 | 7 | 7 | 7 | 7 | 7 |
| R squared | 0.997 | 0.995 | 0.9624 | 0.9932 | 0.9978 | 0.9636 | 0.986 | 0.9966 |
| Sum of Squares | 21.93 | 50.98 | 116.1 | 55.18 | 11.7 | 198.4 | 20.73 | 20.54 |
| Sy.x | 1.77 | 2.699 | 4.072 | 2.808 | 1.293 | 5.324 | 1.721 | 1.713 |

**Table S3.** Anti-drug antibody (ADA) titers against PD1/IL15 TaCk from single dose study

| **Dose** |  | **ADA titers** | | | | |
| --- | --- | --- | --- | --- | --- | --- |
|  | Animal | Pre-dose | Day 6 | Day 14 | Day 21 | Day 28 |
| 0.1 mg/kg | 7001 | <1.70 | 2.35 | 2.14 | 2.39 | 2.14 |
|  | 7002 | <1.70 | 2.49 | 2.45 | 2.22 | 2.10 |
|  | 7003 | <1.70 | 2.45 | 2.52 | 2.61 | 2.53 |
| 0.3 mg/kg | 8001 | <1.70 | 2.25 | 2.04 | 1.81 | <1.70 |
|  | 8002 | <1.70 | 2.64 | 2.56 | 3.52 | 4.34 |
|  | 8003 | <1.70 | 2.93 | 3.60 | 3.43 | 3.84 |
| 1 mg/kg | 9001 | <1.70 | 2.28 | 2.14 | 1.90 | 1.89 |
|  | 9002 | <1.70 | <1.70 | 2.09 | <1.70 | <1.70 |
|  | 9003 | <1.70 | 2.96 | 3.58 | 3.53 | 3.91 |

IL-15 = interleukin 15; TaCk = targeted cytokine; PD-1 = programmed cell death 1 receptor

**Table S4.** Peak (mean) fold expansion of cells after the first dose of PD1/IL15 TaCk in cynomolgus monkeys

|  | **Peak (mean) fold expansion (fold change to baseline) after first dose of PD1/IL15 TaCk** | | | | | | | |
| --- | --- | --- | --- | --- | --- | --- | --- | --- |
|  | Single dose study | | | Non-GLP repeat dose study | | GLP repeat dose study | | |
| **Dose (mg/kg)** | **0.1** | **0.3** | **1** | **0.05** | **0.6** | **0.03** | **0.1** | **0.3** |
| Total CD8^+^ T cells | 4.2 | 11 | 39 | 2.4 | 15 | 1.0 | 3.0 | 8.0 |
| PD-1^+^ CD8^+^ T cells | 9.0 | 19 | 87 | 5.1 | 16.3 | 2.7 | 7.6 | 23.4 |
| Total CD4^+^ T cells | 3.4 | 4.3 | 9 | 1.8 | 4.7 | 1.2 | 2.2 | 3.3 |
| PD-1^+^ CD4^+^ T cells | 9.0 | 11 | 19 | 4.5 | 5.3 | 2.8 | 3.1 | 7.1 |
| DN T cells | 14 | 102 | 257 | 24 | 161 | 10 | 23 | 57 |
| T_regs_ | 20 | 28 | 238 | 3.4 | 51 | 3.5 | 9.5 | 38.5 |
| NK cells | 1.2 | 1.2 | 2.5 | 1.0 | 1.0 | 1.0 | 1.0 | 1.0 |
| CD8^+^ T_CM_ | 5.8 | 6.5 | 29 | 2.4 | 28 | 1.5 | 6.5 | 10.8 |
| CD8^+^T_SCM_ | 9.4 | 29 | 61 | 1.8 | 8 | 1 | 2.8 | 4.2 |
| CD8^+^T_EM_ | 6.5 | 13 | 60 | 1.5 | 27 | 1.5 | 3.8 | 8 |
| CD8^+^T_eff_ | 6.6 | 20 | 110 | 3.3 | 20 | 1.3 | 4.3 | 14.6 |
| CD4^+^ T_CM_ | 6.0 | 7.0 | 12 | 2.0 | 7.0 | 1.8 | 3.8 | 6.2 |
| CD4^+^T_SCM_ | 16 | 29 | 35 | 1.8 | 5 | 1.0 | 2.7 | 6.4 |
| CD4^+^T_EM_ | 9.0 | 14 | 42 | 4.2 | 19 | 1.4 | 4 | 5.4 |
| CD4^+^T_eff_ | 17 | 14 | 32 | 2.0 | 3.0 | 1.0 | 1.5 | 2.3 |

IL-15 = interleukin 15; TaCk = targeted cytokine; PD-1 = programmed cell death 1 receptor; T_CM_ = central memory T cells; T_SCM_ = stem cell memory T cells; T_EM_ = effector memory T cells; T_eff_ = terminal effector T cells; GLP = good laboratory practice

**Table S5.** Flow cytometry immunophenotyping panels

| **Fluorophore** | **Panel 1** | **Panel 4** |
| --- | --- | --- |
| FITC/AF488 | FoxP3 | CD3 |
| PE | CD8b | CD45 |
| PE-CF594/PE Dazzle594 | CD56 | CCR7 |
| PerCP-Cy5.5/PerCP-Vio700 | CD45 | CD20 |
| PE-Cy7/PE-Vio770 | CD8a | CD25 |
| APC/eFluor660/AF647 | PD-1 | CD8b |
| AF700 | Ki67 | CD14 |
| APC-Cy7/APC-H7 | CD45RA | CD45RA |
| BV421/V450/VioBlue | CD3 | CD8a |
| BV510 | CD25 | HLA-DR |
| BV605 | CD14 | CD28 |
| BV650 | CD16 | CD16 |
| BV711 | CD20 | CD95 |
| BV785 | CD4 | CD4 |

**Table S6.** Anti-drug antibody (ADA) titers against PD1/IL15 TaCk from repeat dose non-GLP cynomolgus monkey study

|  | **ADA titers** | | | | | | | | | |
| --- | --- | --- | --- | --- | --- | --- | --- | --- | --- | --- |
| **Dose** | **Animal** | **Pre-dose** | **Day 6*** | **Day 8** | **Day 9** | **Day 14*** | **Day 15, pre** | **Day 23** | **Day 29, pre** | **Day 37, pre** |
| 0.05 mg/kg | 4001 | <1.7 | - | - | 2.61 | - | 3.39 | 4.39 | 4.42 | 4.81 |
|  | 4003 | <1.7 | - | - | 1.88 | - | <1.7 | 2.58 | 3.49 | 4.55 |
|  | 4004 | <1.7 | - | - | <1.7 | - | <1.7 | 2.65 | 3.17 | 4.98 |
|  | 4102 | <1.7 | - | - | 3.08 | - | 3.00 | 2.63 | 2.56 | 2.53 |
| 0.6 mg/kg | 5001 | <1.7 | - | 3.01 | - | - | - | - | - | - |
|  | 5004 | <1.7 | - | - | 2.90 | 2.74 | - | - | - | - |
|  | 5102 | <1.7 | 2.93 | - | - | - | - | - | - | - |
|  | 5103 | <1.7 | - | - | 3.73 | 4.01 | - | - | - | - |

IL-15 = interleukin 15; TaCk = targeted cytokine; PD-1 = programmed cell death 1 receptor; GLP = good laboratory practice; pre = pre-dose

**Table S7.** Anti-drug antibody (ADA) titers against PD1/IL15 TaCk from repeat dose GLP cynomolgus monkey study

| **Dose** | **Day 1, pre** | **Day 8** | **Day 15** | **Day 22,**  **pre** | **Day 29** | **Day 36** | **Day 43,**  **pre** | **Day 50** | **Day 57** | **Day 71** | **Day 106** |
| --- | --- | --- | --- | --- | --- | --- | --- | --- | --- | --- | --- |
| **ADA Titer Range** | | | | | | | | | | | |
| Control | 2.00 | 1.96 | 1.90 | 2.09 | 1.95 | 1.96 | 2.01 | 1.88 | - | - | - |
| 0.03 mg/kg | 3.30 | 1.92–3.26 | 1.47–3.53 | 2.25–4.56 | 1.43–5.32 | 1.89-4.59 | 2.23-5.09 | 2.73-5.05 | - | - | - |
| 0.1 mg/kg | 1.50 | 1.57–3.14 | 1.35–3.46 | 1.53–3.99 | 2.20–5.24 | 1.51-5.42 | 1.95-5.40 | 3.35-6.04 | 2.91-6.18 | 3.15-5.90 | 2.19-5.21 |
| 0.3 mg/kg | - | 2.41–4.60 | 1.81-4.39 | 1.52–4.08 | 1.85–4.91 | 2.14-4.69 | 1.81-4.42 | 1.69-4.28 | 1.74-4.24 | 1.39-3.91 | 1.68-3.26 |

IL-15 = interleukin 15; TaCk = targeted cytokine; PD-1 = programmed cell death 1 receptor; GLP = good laboratory practice; pre = pre-dose

**Table S8.** Peak (mean) fold expansion of cells after the second (non-GLP and GLP study) and third dose (Only GLP study) of PD1/IL15 TaCk in cynomolgus monkeys

| **Peak (mean) fold expansion (fold change to baseline) after first dose of PD1/IL15 TaCk** | | | | | |
| --- | --- | --- | --- | --- | --- |
|  | Non-GLP repeat dose study  **(2^nd^ dose )** | | GLP repeat dose study  **(2^nd^ /3^rd^ dose)** | | |
| **Dose (mg/kg)** | **0.05** | **0.6** | **0.03** | **0.1** | **0.3** |
| Total CD8^+^ T cells | 1.8 | NA | 1.4/1.1 | 2.2/1.6 | 6.1/4.6 |
| Total CD4^+^ T cells | 1.4 | NA | 1.2/1.1 | 1.8/1.3 | 3.3/2.6 |
| DN T cells | 10.8 | NA | 8.9/6.1 | 15.9/4.6 | 23.5/10.6 |
| T_regs_ | 3.2 | NA | 4.2/3.0 | 6.2/5.3 | 60.4/61.9 |
| NK cells | 1.0 | NA | 1.0 | 1.0 | 1.0 |
| CD8^+^ T_CM_ | 1.2 | NA | 1.5/1.7 | 3.2/2.5 | 7.5/7.8 |
| CD8^+^T_SCM_ | 1.6 | NA | 1.7/1.6 | 2.2/1.9 | 4..6/4.1 |
| CD8^+^T_EM_ | 1.0 | NA | 1.5/1.6 | 2.1/1.6 | 9.6/6.1 |
| CD8^+^T_eff_ | 2.5 | NA | 1.9/1.5 | 3.1/1.9 | 11.4/7.1 |
| CD4^+^ T_CM_ | 1.2 | NA | 1.4/1.2 | 2.9/2.2 | 9.1/7.4 |
| CD4^+^T_SCM_ | 1.3 | NA | 1.4/1.0 | 3.3/1.4 | 7.1/4.6 |
| CD4^+^T_EM_ | 1.0 | NA | 1.6/1.0 | 3.3/2.4 | 10/8.2 |
| CD4^+^T_eff_ | 2.2 | NA | 1.3/1.0 | 1.5/2.0 | 5.4/9.6 |

IL-15 = interleukin 15; TaCk = targeted cytokine; PD-1 = programmed cell death 1 receptor; T_CM_ = central memory T cells; T_SCM_ = stem cell memory T cells; T_EM_ = effector memory T cells; T_eff_ = terminal effector T cells; GLP = good laboratory practice

**Supplemental Figures**

| **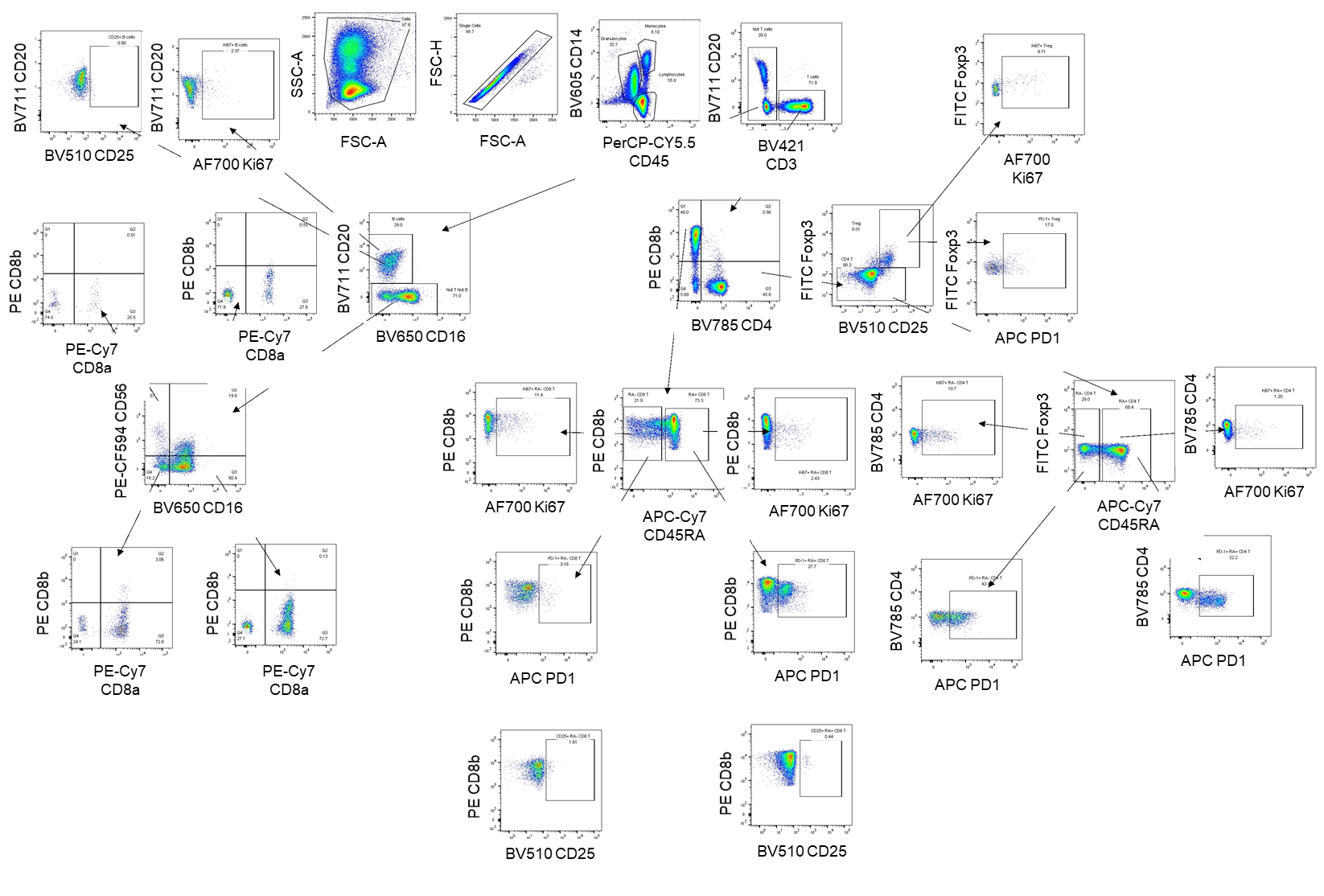**  **(B)** |
| --- |
| **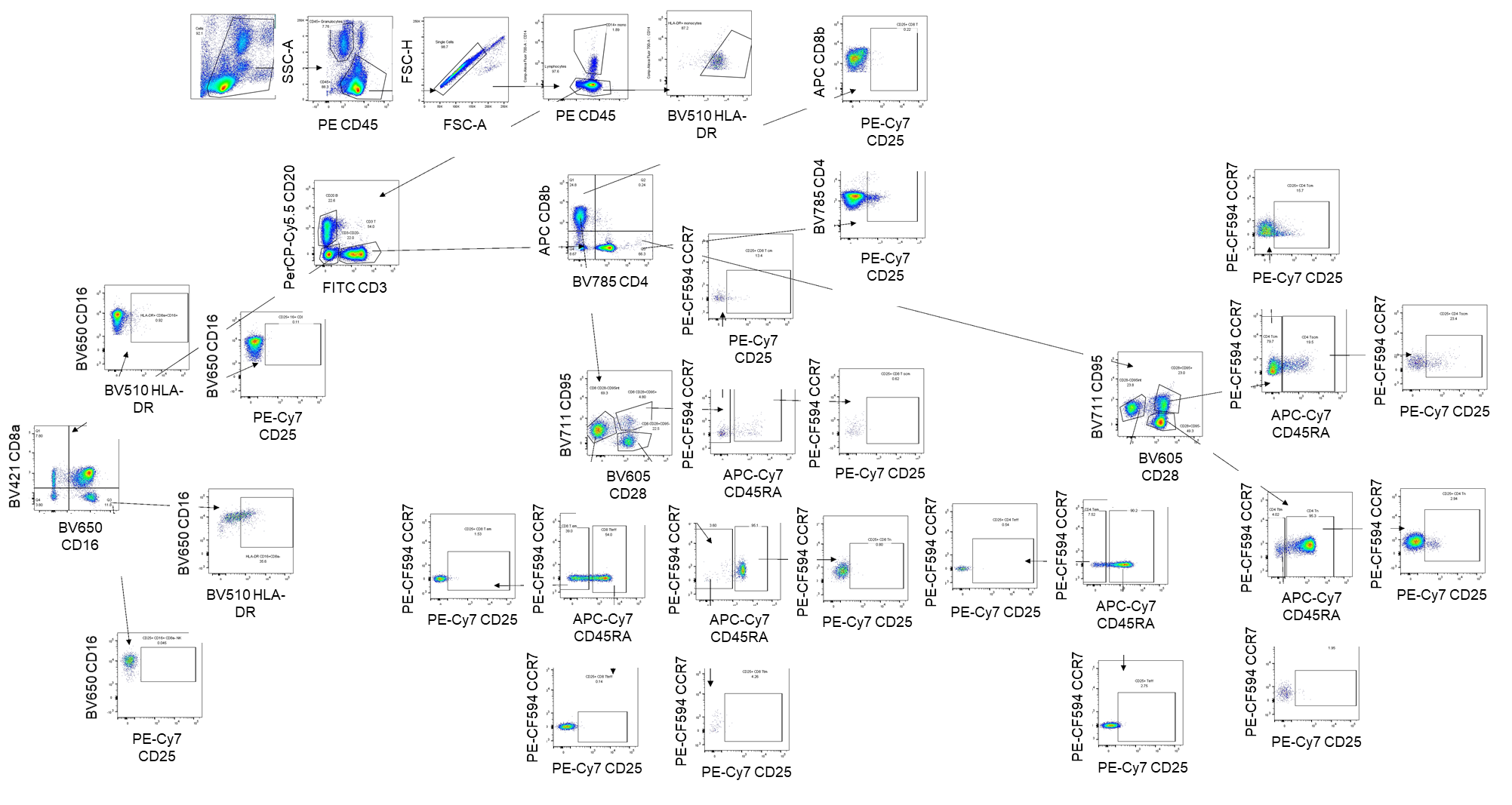** |

**Figure S1:**

**(A)** Antibody (panel 1) gating strategies, intracellular

**(B)** Antibody (panel 4) gating strategies, surface markers

**
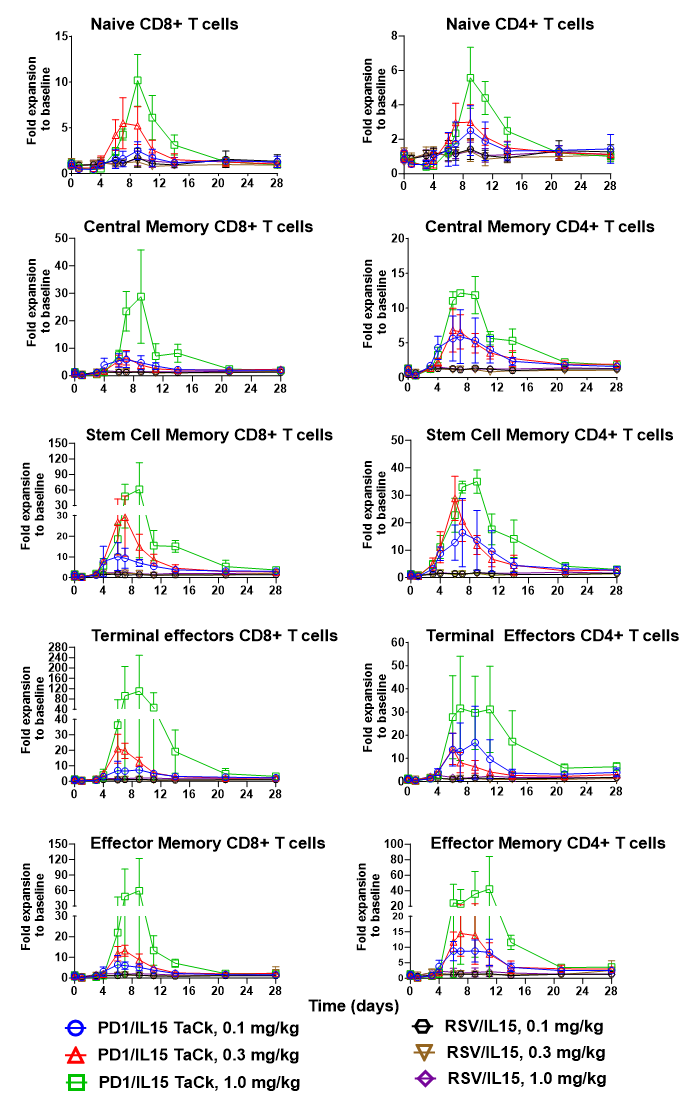
**

**Figure S2.** Change in abundance of PD-1^+^ lymphocytes, including naïve CD8^+^ and CD4^+^ T cells and memory subsets (central memory, stem cell memory, effector memory, and terminal effector phenotype), following a single intravenous bolus administration of PD1/IL15 TaCk in cynomolgus monkeys (n=3/group). Three dose groups were studied: 0.1 mg/kg, 0.3 mg/kg, and 1 mg/kg. The data are represented as mean ± SD

| **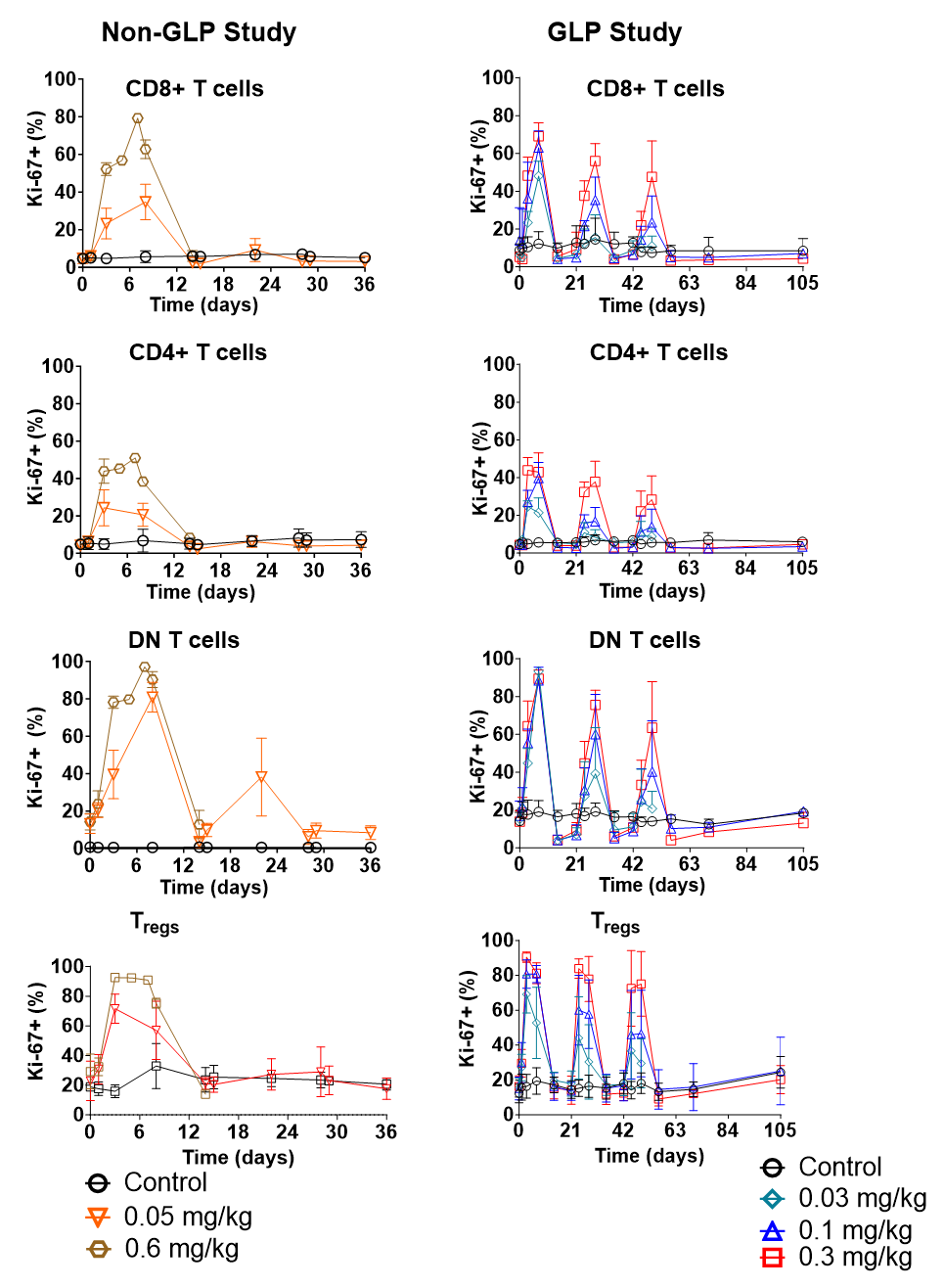** |
| --- |

**Figure S3. Ki67+ data from Non GLP and GLP study**

PD1/IL15 TaCk showed dose-dependent increases in frequencies of Ki-67 detection in CD8^+^, CD4^+^, and CD8^–^CD4^–^ T cell subsets. (**A**) Non-GLP study: Dose-dependent % Ki-67^+^ expression on lymphocytes (CD8^+^, CD4^+^, and CD4^–^CD8^–^ T cells) following repeat IV administration (Q2W) in cynomolgus monkeys (n = 3/group). The data for the 0.6 mg/kg dose group were only available for the first cycle due to unscheduled euthanasia on day 14. (**B**) GLP study: Dose-dependent % Ki-67^+^ on lymphocytes (CD8^+^, CD4^+^, and CD4^–^CD8^–^ T cells) following repeat IV administration (Q3W) in cynomolgus monkeys [In GLP study: n=10 (5M/5F) for control; n=6 (3F/3M) for 0.03 mg/kg; n=10 (5F/5M) for 0.1 mg/kg; n=10 (5F/5M) for 0.3 mg/kg dose groups]. The data are represented as mean ± SD.

**
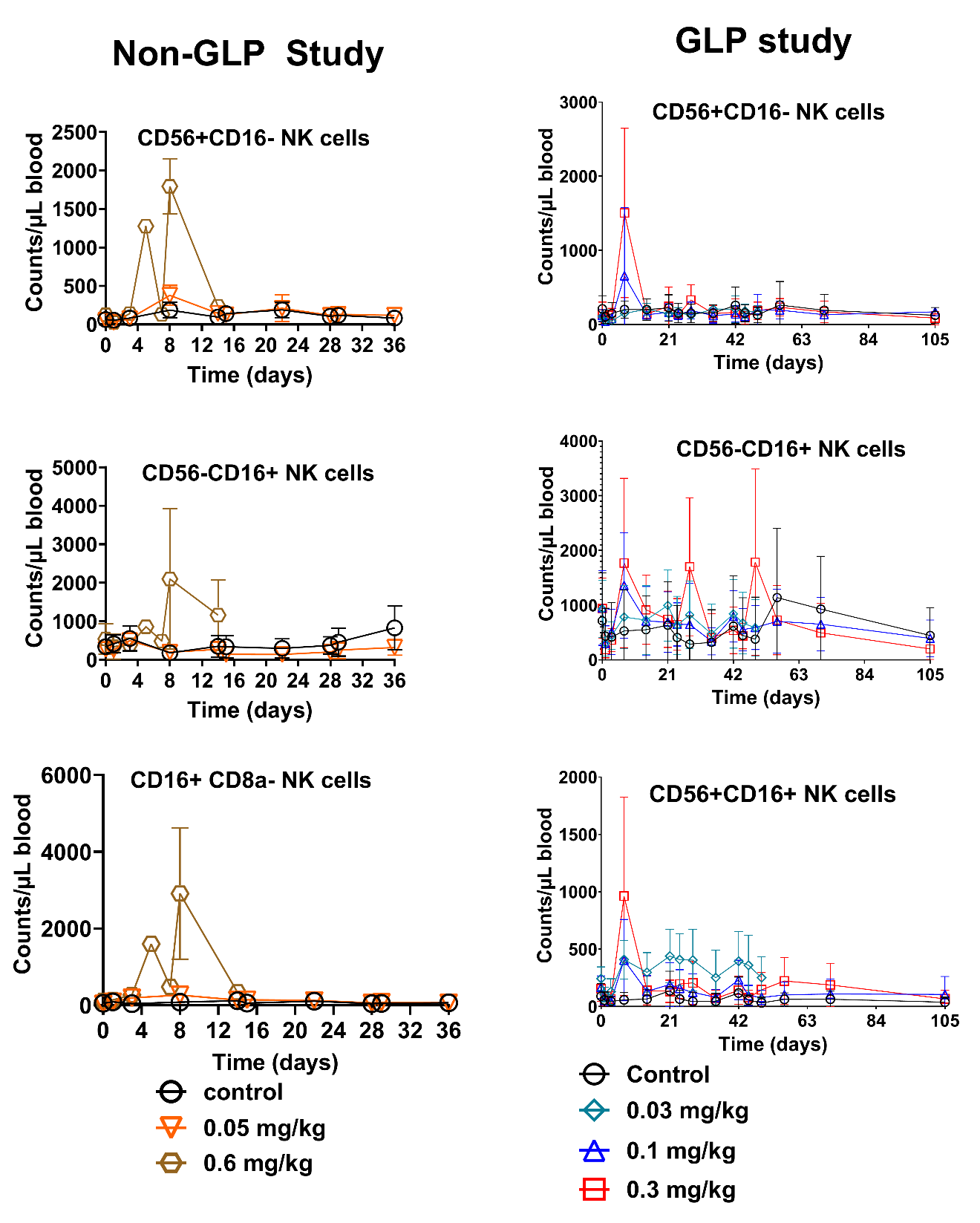
Figure S4:** Repeat dose absolute count profiles of less abundant NK cells from non-GLP and GLP cynomolgus monkey studies. In non-GLP study PD/IL15 TaCk was given Q2W, IV in cynomolgus monkeys (n = 3/group) for three doses. In GLP study: PD1/IL15 TaCk was given Q3W, IV for three doses [n = 10 (5M/5F) for control, n=6; (3F/3M) for 0.03 mg/kg, n=10; (5F/5M) for 0.1 mg/kg, and n=10; (5F/5M) for 0.3 mg/kg dose groups]. The data are represented as mean ± SD.

**Figure S6:** ***In vitro* activity of PD1/IL15 TaCk, on activated CD8^+^ , CD4^+^ and their Effector Memory T cells.** CFSE=carboxyfluorescein succinimidyl ester. Human peripheral blood mononuclear cells (PBMC) were stimulated with 25 ng/mL plate-bound anti-CD3 (OKT3) for 24 h then labeled with CFSE, treated with increasing concentrations of PD1/IL15 TaCk for 4 days at 37 °C, and then analyzed by flow cytometry using CFSE to measure proliferation.
